# Mediating Effect of Heat Waves between Ecosystem Services and Heat-Related Mortality of Characteristic Populations: Evidence from Jiangsu Province, China

**DOI:** 10.3390/ijerph20032750

**Published:** 2023-02-03

**Authors:** Lu Wang

**Affiliations:** Faculty of Civil Engineering and Mechanics, Jiangsu University, Zhenjiang 212013, China; luwang@ujs.edu.cn

**Keywords:** heat wave, mortality, ecosystem services, population health, distributed lag nonlinear model, process analysis

## Abstract

In the context of climate change, heat waves are a serious hazard having significant impacts on human health, especially vulnerable populations. Many studies have researched the association between extreme heat and mortality. In the context of urban planning, many studies have explored the cooling effect of green roofs, parks, urban forests and urban gardens. Nevertheless, few studies have analyzed the effect mechanism of specific ecosystem services (Ess) as mitigation measures to heat waves. This study aimed to determine the relationship among Ess, heat waves and the heat-related mortality risk of different groups by diseases, age and sex. The research was conducted in three cities in Jiangsu Province, including Nanjing, Suzhou and Yancheng. We quantified five ecosystem services, i.e., water supply service, carbon sequestration service, cooling service, biodiversity and cultural service. Based on the previous studies, we took the frequency of heat waves into account, extending the concept of the Heat Wave Magnitude Index (HWMI). A distributed lag nonlinear model (DLNM) was applied to estimate the effect of extreme heat on mortality. Then, the study used the process analysis method to explore the relationship among Ess, heat waves and heat-related mortality risks. The results indicated that (i) water supply service, carbon sequestration service, cooling service and biodiversity can reduce heat-related mortality while cultural service increases; (ii) the effects of carbon sequestration service and cultural service are stronger than other Ess; (iii) the effects of Ess on cardiorespiratory disease, stroke and chronic obstructive pulmonary disease (COPD) mortality risks are higher than others; and (iv) women and elderly heat-related mortality risks are more affected by the Ess. This study can provide a theoretical support for policy makers to mitigate heatwave events, thus limiting heat-related mortality.

## 1. Introduction

Currently, climate change is a paramount challenge, and almost all regions of the world are experiencing warming [1,2]. There is a general agreement that extreme heat affects the body’s temperature-regulating mechanism, leading to heat-related diseases and exacerbating cardiorespiratory diseases [3]. Heat waves are a particular type of extreme heat that are defined as prolonged periods of excessively high temperatures beyond human adaptation [4]. Global average surface temperatures are projected to increase by 1.1–6.4 °C by 2100, and heat waves are also increasing in frequency, intensity and duration [5]. Several studies [6,7,8] have reported that extreme heat influences mortality, making it a current public health concern. Heat waves are associated with increased mortality due to nonaccidental and specific causes [9]. Previous studies have identified the differences in the impact of mortality on different sexes and age groups [10,11]. The findings on populations vulnerable to heat waves are somewhat inconsistent [12]. Most studies have suggested that the elderly and women are more vulnerable to extreme heat due to physiological and social characteristics [10,13,14]. However, studies from China and Sweden indicated that men are more affected by heat waves than women [15,16]. Additionally, young adults may be even more vulnerable to heat waves because they are more likely to engage in strenuous outdoor work and activities during heat waves [15].

A series of factors can modify the effects of heat on human health, such as sex, the division of labor and exposure to extreme heat and age [17,18]. The role of sex as a risk factor remains unclear, and the assessment is limited [17]. Hence, achieving a better understanding of the heterogeneity of the sexes in different age groups is essential to identify vulnerable populations exposed to heat. Under the context of climate change, the increasing severity of heat waves has inflicted a disastrous effect on public health [19]. Adaptation and mitigation strategies are needed to reduce heat-related mortality risks.

Both politics and academia are pushing toward the implementation of nature-based solutions (NBSs), defined as “actions which are inspired by, supported by or copied from nature”, which can provide multiple ecosystem services (Ess) [20]. Ess have become increasingly important over the last 15 years [21] and are defined as “the benefits that people get from ecosystems” [22]. Indeed, some studies have pointed to the relationships between Ess in regulating the local thermal environment [23,24,25,26]. Planting trees and deploying green infrastructure will help to cool areas and mitigate the effects of natural disasters [20]. Trees and vegetation can lower summer temperatures by providing shade, causing evapotranspiration and removing greenhouse gases [25,26]. Cultural service may have a positive correlation with the thermal environment, as the increase in cultural service may lead to a decrease in cooling service. It is essential to highlight that Ess significantly affect the thermal environment and further impact heat-related health.

Although a number of studies have assessed the effect of Ess on heat-related mortality, the results are inconsistent. For example, Zhang et al. [27] revealed that greenness can protect against the effect of heat waves on mortality; Burkart et al. [28] found that urban green and blue appeared to have a mitigating effect on heat-related mortality in the elderly population in Lisbon. Marvuglia et al. [29] found that green roofs act to lower the damaging effects of heat waves on human health. Choi et al. [30] explored the effect modification of greenspace on the heat-mortality relationship on a global scale. Nevertheless, some studies pointed out that green space is not associated with heat-related mortality [31,32]. The effect mechanism of Ess on heat waves and heat-related mortality remains to be studied.

Therefore, taking three different cities of Jiangsu Province as the study area, the main objectives of this study were: (i) to determine the populations vulnerable to extreme heat, (ii) to analyze how Ess affect heat waves and heat-related mortality, and (iii) to compare the effects of different Ess on human health. This study quantified the annual magnitude of heatwave events and five ecosystem services from 2000 to 2015 in Jiangsu Province. Then, three cities (Nanjing, Suzhou and Yancheng) in Jiangsu Province were selected to evaluate the mortality risk from exposure to extreme heat from 2007 to 2015. For the three cities, this study derived the Ess, HWMI and RRs from 2007 to 2015 to explore the relationship among Ess, heat waves and mortality risk.

Addressing these issues will be beneficial for revealing the effect mechanism of Ess on heat waves and human health; these findings will provide a theoretical support for urban planning to reduce the risk of thermal impacts and identify the most vulnerable populations.

## 2. Materials and Methods

### 2.1. Study Area

**Figure 1:**
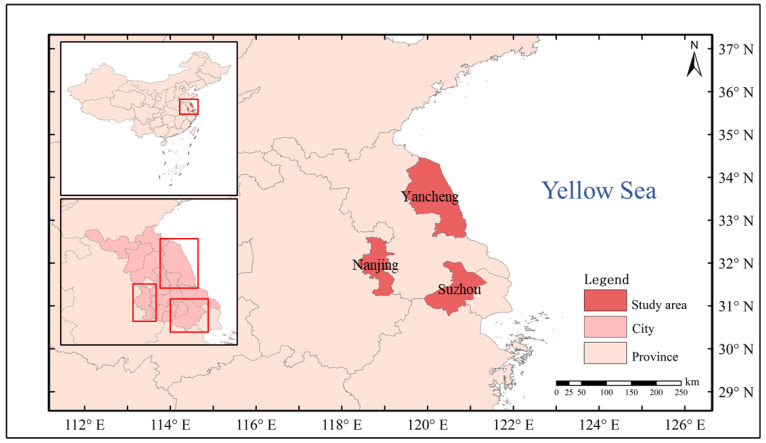
Location of the study area.

This study was conducted in three cities (Nanjing, Suzhou and Yancheng) with different latitudes in Jiangsu Province, China (Figure 1). These three cities included a total of 25.8 million people in 2010. Jiangsu Province is one of the more developed provinces in China and has experienced rapid urbanization [33]. Nanjing (31°14′ N–32°37′ N, 118°22′ E–119°14′ E), the capital of Jiangsu Province, is located centrally within the province. Nanjing has a northern subtropical, humid monsoon climate. From 2007 to 2015, in Nanjing, the annual average temperature was 16.4 °C, the annual precipitation was 1201.6 mm, the average temperature of the coldest month (January) was 3.0 °C and the average temperature in the hottest month (July) was 28.3 °C [34]. Suzhou (30°47′ N–32°02′ N, 119°55′ E–121°20′ E), located in the south of Jiangsu Province, is characterized by extensive rural industrialization and dramatic economic growth. From 2007 to 2015, the annual average temperature was 17.3 °C, the annual precipitation was 1170.2 mm, the average temperature of the coldest month (January) was 4.3 °C and the average temperature in the hottest month (July) was 29.3 °C. Nanjing and Suzhou lie along the Yangtze River. Yancheng (32°48′ N–34°29′ N, 119°53′ E–121°18′ E) is in the northern coastal area of Jiangsu Province and has an integral ecosystem and high habitat value. Yancheng has a subtropical monsoon climate, whereas Suzhou has a subtropical monsoon marine climate. From 2007 to 2015, in Yancheng, the annual average temperature was 15.0 °C, the annual precipitation was 1025.5 mm, the average temperature of the coldest month (January) was 1.5 °C and the average temperature in the hottest month (July) was 27.3 °C.

### 2.2. Data Collection

The data used in this study are shown in Table 1. Daily data on maximum temperature, relative humidity and wind speed were obtained from weather stations in each city. Daily cause-specific mortality data from 1 January 2007 to 31 December 2015, were collected from the Jiangsu Provincial Center for Disease Prevention and Control. Daily mortality counts were classified into nonaccidental (A00-R99) and cardiorespiratory mortality (I00-I99, J00-J99) based on the International Statistical Classification of Diseases and Related Health Problems, 10th Revision (ICD-10). Cardiorespiratory diseases were subdivided into hypertensive diseases (I10-I15), IHD (I20-I25), stroke (I60-I69) and COPD (J40-J47). This study also investigated the effect of heat on cardiorespiratory mortality modified by sex (men and women) and age (15–64, 65–74 and 75+).

### 2.3. Quantifying Ecosystem Services

#### 2.3.1. Water Supply Service

Water supply is defined as the amount of water resources available to humans and is calculated as the difference between precipitation and actual evapotranspiration. The annual water yield per grid is calculated as follows [35]:

(1) WY=P−AET=(1−AET/P)×P

AET/P is calculated as follows [36]:

(2) $$\mathrm{AET}/P=\left(1+\omega R\right)/\left(1+\omega R+R^{-1}\right)$$

where WY refers to the water yield; P is the annual precipitation; AET is the actual evapotranspiration; R is the ratio of potential evapotranspiration to precipitation; and the ω factor is the plant-available water coefficient, which is a dimensionless parameter.

For this study, the plant-available water coefficient was assumed to be 2.0 for woodland, 1.0 for shrubland, 0.5 for grassland and cropland, and 0.1 for construction land and unused land [37]. The ET of a water body is defined as the minimum precipitation and potential evapotranspiration [38].

#### 2.3.2. Carbon Sequestration Service

Plants can absorb carbon dioxide through photosynthesis. Based on the photosynthesis equation, $6{\mathrm{CO}}_{2}+6H_{2}O\to 6O_{2}+C_{6}H_{12}O_{6}$, net primary production (NPP) was used to calculate carbon sequestration, where the production of 1 kg of organic matter can fix 1.63 kg of $CO_{2}$ [39,40].

(3) CS=NPP×1.63

where CS refers to carbon sequestration.

#### 2.3.3. Cooling Service

The normalized difference vegetation index (NDVI) is a vegetation index based on satellite images. Values around zero represent bare soil, and higher values indicate the higher levels of vegetation density. The cooling power of vegetation has been widely proved and green space has been proposed as a natural mitigation strategy for climate change [41]. Trees and vegetation can modulate the thermal environment by providing shade and transpiration [25]. Thus, the mean NDVI value was used to assess the cooling services of each city in this study.

#### 2.3.4. Biodiversity

The spatial arrangement and combination of land surface elements could impact ecological functions and processes. Animals can travel more easily in habitats with low resistance. A lower patch connectivity indicates that animals must overcome greater migration resistance [42]. Thus, the study used the patch cohesion index (COHESION) to assess biodiversity, which represents the connectivity of habitats. COHESION was calculated as follows:

(4) $$\mathrm{COHESION}=\left[1-\frac{\sum_{j=1}^{n}P_{ij}}{\sum_{j=1}^{n}P_{ij}\sqrt{a_{ij}}}\right]\times {\left[1-1/\sqrt{N}\right]}^{-1}\times 100$$

where $P_{ij}$ is the perimeter of patch ij in terms of the number of cell surfaces; $a_{ij}$ is the area of patch ij in terms of the number of cells; and Z is the total number of cells in the landscape.

#### 2.3.5. Cultural Service

Landscape metrics as descriptions of landscape patterns are believed to have a relationship with climate and are crucial for the maintenance of both cultural diversity and biodiversity [43,44,45]. Shannon’s diversity index (SHDI) was used to represent landscape diversity and assess cultural services [46]. SHDI was calculated as follows:

(5) $$\mathrm{SHDI}=-\overset{m}{\underset{i=1}{\sum }}\left(P_{i}\times lnP_{i}\right)$$

where *I* indicates the land cover type; $P_{i}$ is the proportion of the landscape occupied by land cover type I; and m indicates the number of types.

In this study, the WY, CS and NDVI from 2000 to 2015 were estimated by using ArcGIS 10.7 software to analyze the temporal and spatial changes. The COHESION and SHDI from 2000 to 2015 were calculated by using Fragstats 4.2 software.

### 2.4. Magnitude of Annual Heatwave Events

In this study, heat waves are defined as ≥3 consecutive days with daily maximum temperature >90th percentile [19]. Heat waves within a given year were examined with the Heat Wave Magnitude Index (HWMI). HWMI is calculated by summing of all heatwave magnitudes in this year. Heatwave magnitude is defined as the sum of the magnitudes of all subheat waves that comprise a heat wave [47]. A subheat wave is a heat wave of three consecutive days. The multiple-stage process is explained as follows [47]:

(1) Daily threshold: In this study, the threshold is the 90th percentile of the daily maximum temperature from 2000 to 2015.
(2) Heatwave selection: The periods with an excessive threshold for three days or more are selected as heat waves.
(3) Heat wave to subheat waves: Each heat wave can be decomposed to n subheat waves. A subheat wave is three consecutive heat wave days. For example, if the length of a detected heat wave is 11 days, then the study obtained 3 subheat waves for a total of 9 days; the last 2 days of the heat wave are grouped with the value below the threshold. Thus, the last subheat wave of the heat wave includes 3 days as well.
(4) Subheat wave magnitude: The maximum temperatures for three consecutive days are added together to obtain the unscaled magnitude. The subheat wave unscaled magnitude is normalized from 0 to 1 by the min–max normalization method.
(5) Heatwave magnitude: The heatwave magnitude is defined as the sum of n subheat wave magnitudes.
(6) Heat Wave Magnitude Index: The HWMI is defined as the sum of all heatwave magnitudes in this year.

### 2.5. Mortality Risk Caused by Heat

A distributed lag nonlinear model (DLNM) with a quasi-Poisson regression was applied to investigate the relationship between daily maximum temperature and nonaccidental and cause-specific mortality by age and sex in each city. Consistent with previous studies [48,49], the exposure to extreme heat was defined as the 97.5th percentile of temperature distribution relative to the MMT in this study. Firstly, this study evaluated the exposure–response relationship between temperature and mortality from 2007 to 2015 using the DLNM. Secondly, the study calculated the 97.5th percentile of temperature for the entire period and each year. Thirdly, this study calculated the RRs of the entire period and for each year based on the exposure–response relationship between temperature and mortality. The annual RRs were then used to conduct the process analysis of the mediating effect. Additionally, the RRs for individual population groups were calculated in the same way. The DLNM can flexibly describe the nonlinear exposure–response association and simultaneously represent the lagged effect [50]. The model is presented below:

(6) $$\mathrm{LnE}\left(\mathrm{Yt}\right)=\alpha +\varepsilon \mathrm{Cb}.{\mathrm{temp}}_{l}+\mathrm{ns}\left(\mathrm{time},\mathrm{df}\right)+\mathrm{ns}\left(\mathrm{RH},\mathrm{df}\right)+\delta \mathrm{DOW}+{\beta \mathrm{HW}}_{t}$$

where E(Yt) is the expected daily mortality count at day t; β, ε and δ are the coefficients for ${\mathrm{HW}}_{t}$, $\mathrm{Cb}.{\mathrm{temp}}_{l}$ and DOW; RH refers to relative humidity; ${\mathrm{HW}}_{t}\;$is a binary variable; $\mathrm{Cb}.{\mathrm{temp}}_{l}$ is a cross-basis matrix for the two dimensions of temperature and lags; l refers to the maximum lag days; the natural cubic spline function ns( ) captures the nonlinear relationships between the covariate (time and RH) and mortality; DOW is the dummy variable for the day of the week; and α is the intercept.

The main model included a cross-basis function of daily temperature, which included a quadratic B spline with three internal knots placed at the 10th, 75th and 90th percentiles of temperature distributions; a lag response curve with a natural cubic B spline and three internal knots placed at equally spaced values in the log scale with a maximum lag up to 6 days [51], which was used because previous studies showed that the heat effect usually lasted for a week [15,52]; a binary variable which is equal to 1 for heatwave days and 0 for non-heatwave days; 5 degrees of freedom (df) per year for long-term time trends and 3 df for RH, which were applied to the analysis; and a dummy variable for the day of the week. The minimum mortality temperature (MMT) was used as the reference value for calculating the relative risk. The RR corresponding to the 97.5th percentile of temperature in each city was calculated as the mortality risk caused by extreme heat. Several sensitivity analyses were conducted to examine different df for time and different maximum lag days. All analyses were conducted with the R software (version 3.6.1) using the “dlnm” package.

### 2.6. Statistical Analysis

This study derived the Ess, HWMI and RRs of three cities (Nanjing, Suzhou and Yancheng) for each year between 2007 and 2015. Then, the correlation analysis, as a preliminary analysis, was used to explore how the Ess impact heat waves and heat-related mortality This study used the correlation analysis to study the relationship between the two. Pearson’s correlation coefficient can measure the correlation between two variables, *x* and *y* [53]. The coefficient r is calculated as follows:

(7) $$r=\frac{\sum_{i=1}^{n}\left(x_{i}-\bar{x}\right)\left(y_{i}-\bar{y}\right)}{\sqrt{\sum_{i=1}^{n}{\left(x_{i}-\bar{x}\right)}^{2}\sum_{i=1}^{n}{\left(y_{i}-\bar{y}\right)}^{2}}}$$

**Figure 2:**
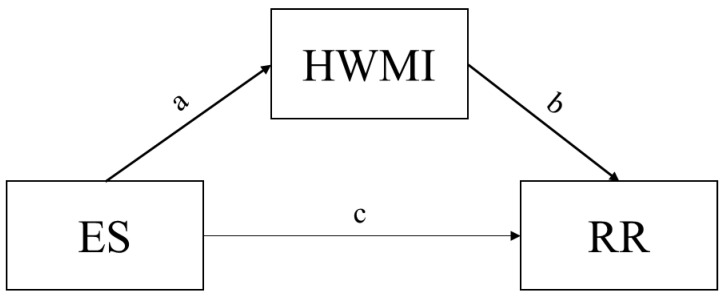
The mediating effect model in this study.

The process analysis method was then utilized to explore the effect mechanism of the Ess, HWMI and RRs, which is an in-depth analysis [54]. The process of mediation is a causal explanation, which assumes that the mediator is causally located between an independent variable (X) and a dependent variable (Y) [55]. The mediating effect refers to how the effect of X on Y is realized by the mediating variable (M) [56]. The model of the mediating effect is shown in Figure 2. In this study, the independent variable (X) is the Ess, the mediating variable (M) is the HWMI and the dependent variable (Y) is mortality risk. The coefficients a, b and c are the path coefficients of the mediation effect model. Coefficient a is the direct effect of X on M, coefficient b is the direct effect of M on Y, and coefficient c is the direct effect of X on Y. The test of joint significance (TJS) was used to test the mediating effect in this study. This approach requires that both the direct effects of X on M and M on Y be statistically significant, and it does not test the total effect and direct effect of X on Y. The mediating effect analysis was conducted in PROCESS, a plug-in for SPSS 25.0.

## 3. Results

### 3.1. Spatial-Temporal Patterns of Ecosystem Services

The spatial–temporal patterns of the ecosystem services in Jiangsu Province from 2000 to 2015 are shown in Figure S1. According to the results in 2000, water supply service, carbon sequestration service and culture service showed an increasing trend in Jiangsu Province, whereas cooling service and biodiversity showed a declining trend. From southeast to northwest, two ecosystem services, i.e., water supply service and carbon sequestration service, display a high-to-low trend; the other three ecosystem services, i.e., cooling service, biodiversity and cultural service, exhibit heterogeneity between the north and the south. Small values of the NDVI and COHESION are mainly observed in the southern areas, which experienced a more rapid urbanization process [57]. In contrast to COHESION, the largest SHDI value was found in the southern area of Jiangsu Province, where developed urban areas lie. Specifically, the vegetation coverage area and connectivity of habitats has decreased due to the process of urban expansion and development.

**Figure 3:**
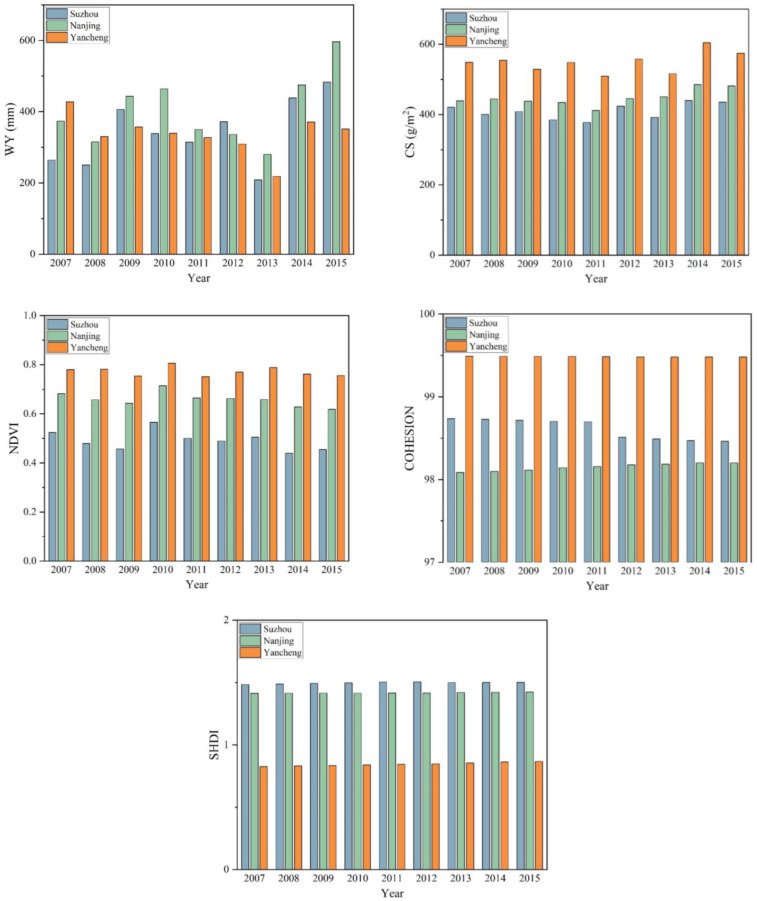
Temporal variation in the five ecosystem services.

The trends of ecosystem services from 2007 to 2015 In Nanjing, Suzhou and Yancheng are shown in Figure 3. The water supply service of Nanjing and Suzhou showed a trend of fluctuation and rise. Compared with 2007, the water supply service of Yancheng decreased in 2015. Yancheng has the strongest carbon sequestration service and cooling service, followed by Nanjing and Suzhou. The biodiversity of Suzhou showed a downward trend, whereas that of Nanjing showed an upward trend. Additionally, biodiversity in Yancheng is relatively stable. Suzhou has the highest cultural service, whereas Yancheng has the lowest.

### 3.2. Spatial-Temporal Patterns of HWMI

**Figure 4:**
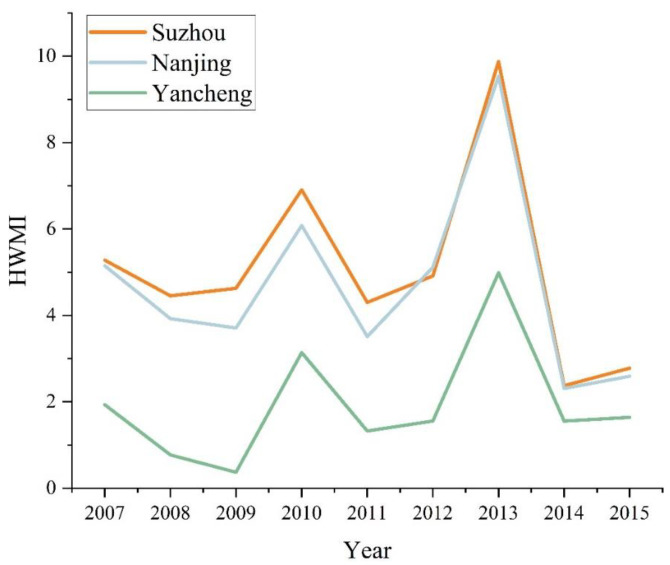
HWMI from 2007 to 2015.

Spatial–temporal patterns of the HWMI in Jiangsu Province are shown in Figure S2. According to the spatial–temporal patterns of the HWMI, heat waves in the north and coastal areas are less severe than those in the south. Jiangsu Province had the highest HWMI in 2013. The decline in the HWMI in 2015 may have been affected by atmospheric circulation. From 2000 to 2013, the high-grade heatwave areas tended to spread from south to north. For the HWMI of Nanjing, Suzhou and Yancheng, Suzhou increased the most, followed by Nanjing and Yancheng. Heatwave events in Suzhou and Nanjing are significantly more severe than in Yancheng. Besides latitude, discrepancies between the three cities may also be affected by rapid urbanization. The trends of the HWMI in Nanjing, Suzhou and Yancheng from 2007 to 2015 are shown in Figure 4. The HWMI of the three cities showed similar trends. The HWMI in Nanjing and Suzhou was higher than that in Yancheng. Additionally, the heat waves were most severe in 2010 and 2013.

### 3.3. Mortality Risks Associated with Extreme Heat

**Figure 5:**
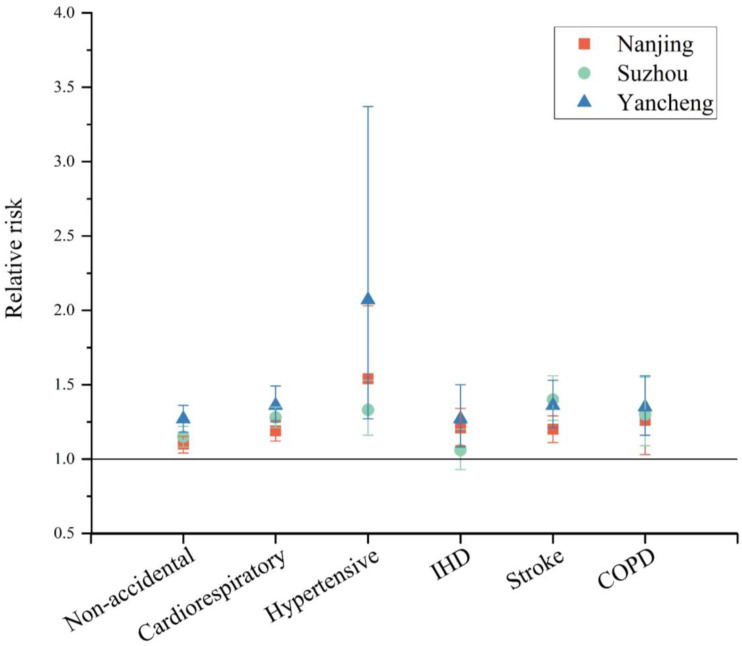
Mortality relative risk stratified by cause of death.

In this study, the mortality risk was expressed as the relative risk (RR) with a 95% confidence interval (CI). RRs of nonaccidental mortality, all cardiorespiratory diseases, hypertensive diseases, ischemic heart disease (IHD), stroke and chronic obstructive pulmonary disease (COPD) in the three cities are shown in Figure 5. The effect of heat on cause-specific mortality in different cities is heterogeneous. The mortality risk in Yancheng is generally greater than in Nanjing and Suzhou. The mortality risks were higher in hypertensive diseases and COPD in Nanjing (RR, 1.54; 95% CI, 1.16–2.03; and RR, 1.26; 95% CI, 1.03–1.55, respectively) and hypertensive diseases and stroke in Suzhou (RR, 1.33; 95% CI, 1.16–1.53; and RR, 1.40; 95% CI, 1.26–1.56, respectively) and Yancheng (RR, 2.07; 95% CI, 1.27–3.37; and RR, 1.36; 95% CI, 1.21–1.53, respectively). The reference temperature used to derive the RR is shown in Tables S3 and S4. Additional sensitivity analyses showed that the results remained similar (Tables S5 and S6). Additionally, the annual mortality risk of the three cities is shown in Figure S3.

**Figure 6:**
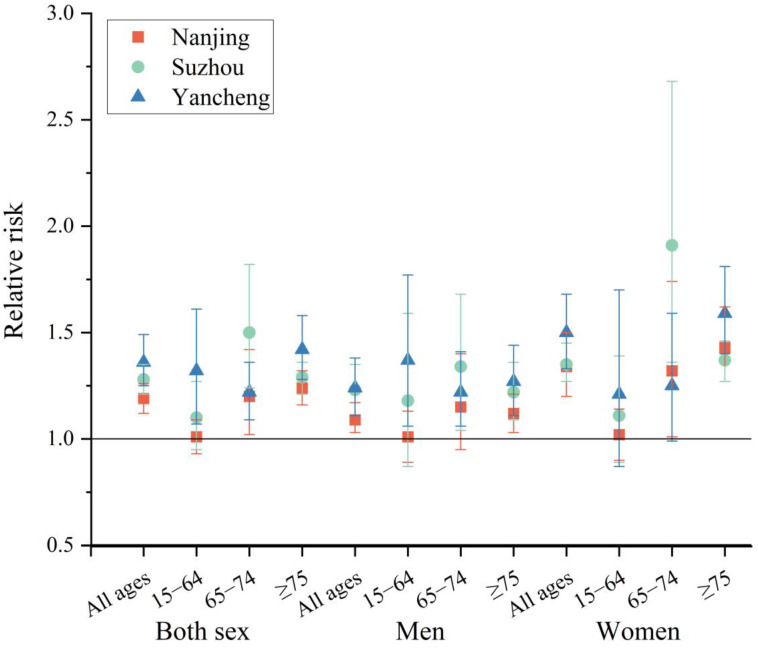
Cardiorespiratory mortality relative risk stratified by sex and age.

The mortality risks of cardiorespiratory disease for different sex and age groups are shown in Figure 6. The results showed that age and sex factors can modify the mortality risks. A positive association between heat and cardiorespiratory mortality was observed for both men and women, and the effect was stronger among women than men in all ages. The mortality risks of sex and age showed heterogeneity in different cities. The mortality risk was highest in the oldest age group (≥75 years) in Nanjing (RR, 1.24; 95% CI, 1.16–1.32) and Yancheng (RR, 1.42; 95% CI, 1.28–1.58), whereas the age group of 65–74 years was affected the most in Suzhou (RR, 1.29; 95% CI, 1.21–1.36). In the age group of 15–64 years, the effect of extreme heat is stronger among men than women in Suzhou and Yancheng. This might be due to outdoor activities and the occupational division of labor [17].

### 3.4. Relationships between ESs, Heat Waves and Heat-Related Mortality Risks

#### 3.4.1. Correlation Analysis

The results of the correlation analysis are listed in Tables S8–S10. Based on the correlation analysis of 23 indicators, statistically significant negative correlations are detected in the WY, CS, NDVI and COHESION with the HWMI, and the effect is strongest on the CS. The SHDI is positively correlated with the HWMI. The results show that the WY and SHDI are negatively correlated with mortality risks. Most mortality risks are positively correlated with the CS, NDVI and COHESION. The CS and NDVI are negatively correlated with mortality risk for people aged 65 to 74. The related mortality risks are also affected by other factors, such as health services, education level, socioeconomic structure, etc. Rapid urban development improves urban public services but reduces ecosystem services [40]. Mortality risks may be offset by the increased socioeconomic level. Thus, this study observed some positive correlations between mortality risks and the ESs such as the CS, NDVI and COHESION.

#### 3.4.2. Mediating Effect Analysis

**Figure 7:**
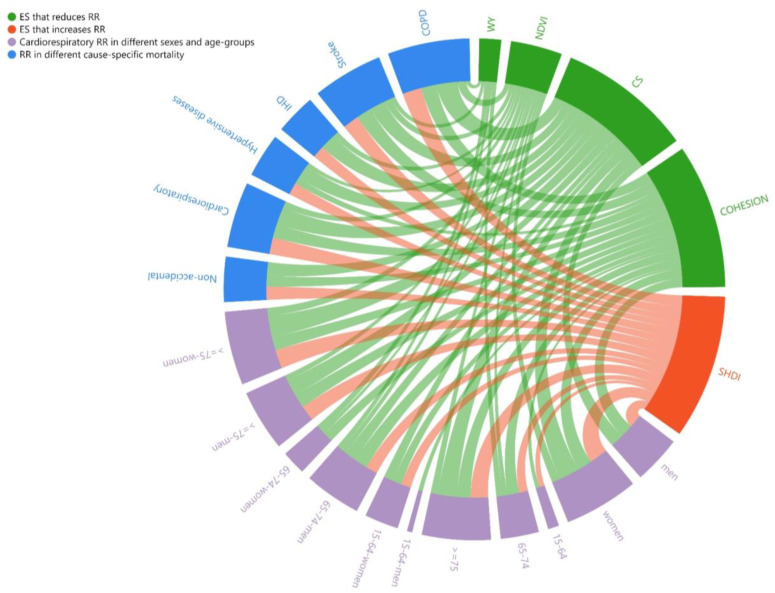
The mediating effects of ESs on mortality risks.

The study focused on the mediating effect of the HWMI between ESs and mortality risk. This study wanted to explore how ecosystem services affect mortality risk by influencing heat waves. Tables S11 and S12 show the specific pathways of how ESs affect cause-specific mortality RR and different groups of cardiorespiratory mortality RR, respectively. The numbers in Tables S11 and S12 refer to the size of the effect of ESs on the HWMI (a) and the direct effect of the HWMI on RR (b). The WY, CS, NDVI and COHESION have an inhibitory effect on the HWMI, thus limiting the heat-related health risks. However, the SHDI has a positive effect on the HWMI and mortality risks. The results show that the effect of CS on the HWMI is strongest, followed by the SHDI, COHESION, NDVI and WY. The results of the mediating effects are shown in Figure 7, and the thickness of a line represents the size of the mediating effect. Table 2 lists the results of the mediating effects (a×b) from Figure 7. The numbers indicate the size of the effect of ESs on RR through affecting HWMI. Generally, the effects on cardiorespiratory disease, stroke and COPD are stronger than on other cause-specific diseases. The mortality risks for the elderly and women are more affected by ESs.

## 4. Discussion

This study analyzed the effect mechanism of ESs on heat waves and heat-related mortality. The results provide direct evidence that ESs have a significant impact on the HWMI, and heatwave events are associated with increased mortality risks of cardiorespiratory diseases. The mortality risks for the elderly and women are more affected by heat waves. This study assessed the associations among ESs, the HWMI and heat-related mortality from a spectrum of causes, and further estimated the cardiorespiratory mortality for different sexes and age groups. The mediating effects of carbon sequestration service and cultural service were higher than other ESs. Additionally, the mediating effects were stronger in cardiorespiratory diseases, the elderly and women. For the subtypes of cardiorespiratory diseases, the effect is stronger on stroke and COPD mortality risks.

The results show that heat waves in Nanjing and Suzhou are more severe than those in Yancheng. This discrepancy may be affected by latitude and urbanization. It is understandable that heat waves are more likely to occur at lower latitudes than at higher latitudes. Urbanization will change the surface physical properties and lead to the formation of the urban heat island (UHI) effect [58]. Synergistic interactions between the UHI effect and heat waves may worsen the thermal environment.

This study found that extreme heat was significantly associated with an increased risk of mortality from nonaccidental and cardiorespiratory diseases. The findings on cause-specific mortality risk showed that people suffering from cardiorespiratory diseases are vulnerable. The study estimated stronger mortality risks for cardiorespiratory diseases such as hypertensive diseases, stroke and COPD compared to the total mortality in each city. The findings are generally consistent with many previous studies [48,51]. The estimated RRs associated with extreme heat (Nanjing, nonaccidental: 1.10, cardiorespiratory disease: 1.19, hypertensive disease: 1.54, IHD: 1.21, stroke: 1.20, and COPD: 1.26; Suzhou, nonaccidental: 1.15, cardiorespiratory disease: 1.28, hypertensive disease: 1.33, IHD: 1.06, stroke: 1.40, and COPD: 1.30; Yancheng, nonaccidental: 1.27, cardiorespiratory disease: 1.36, hypertensive disease: 2.07, IHD: 1.27, stroke: 1.36, and COPD: 1.35) were similar to those in a previous study based on 272 cities in China, which reported a RR of 1.16 for nonaccidental diseases, 1.22 for cardiovascular disease, 1.36 for respiratory disease, 1.24 for stroke and 1.26 for COPD [51]. Ma et al. [48] reported an RR of 1.77 for hypertensive disease. Respiratory symptoms can be linked to the thermoregulatory response and, possibly, the direct effect of breathing hot air, which can lead to increased airway resistance and bronchoconstriction [10]. The prevalence of cardiorespiratory diseases (such as hypertensive disease, IHD, stroke and COPD) may increase the susceptibility to heat waves due to the increased load on the circulation system that is needed to maintain a normal body temperature [6].

The mortality risks for different cities, ages and sexes were heterogeneous. The mortality risk in Yancheng was higher than in Nanjing and Suzhou. The three cities have different climate types [59]. Nanjing has a northern subtropical humid climate. Suzhou has a subtropical monsoon maritime climate. Yancheng has a subtropical monsoon climate. Additionally, the heterogeneity of ecosystem services may be caused by the different climatic characteristics of the three cities. In comparison with people residing in southern regions, those from northern regions may not have awareness and be prepared for extremely high temperatures, and thus, will suffer from a higher detrimental health impact of extreme heat [7]. This difference could reflect adaptation responses and local heat intervention policies such as medical service preparation and heat-protection advice and, consequently, could result in diverse human responses to heat waves in different cities [60]. The study then researched the mortality risks of cardiorespiratory diseases by sex and age. The results showed that women and the elderly had a higher mortality risk. The risk due to heat waves depends on the socioeconomic status of the population, sex and age, as well as the exposure and vulnerability of the population to the heatwave hazard, especially for those who work outside [18]. The RR was significantly elevated in both sexes, and women appear to be more susceptible than men in each city. The greater vulnerability of women has been noted in many previous studies [14,61]. It may be linked to physiological differences in thermoregulation between sexes [14]. The susceptibility of females may be caused by their relatively low ability to adapt to heat in relation to a disadvantaged socioeconomic status [61]. However, the effect varies across age groups and cities. In this study, women have a higher RR than men in the age group ≥ 65 years. Men have a higher RR than women in the age group of 15–64 years in Suzhou and Yancheng. This might be due to outdoor activities and the occupational division of labor. Men are more likely to do outdoor work, which is more exposed to heat. The stronger effect on women could also be partly explained by the fact that women generally live longer than men. Additionally, aged people are particularly vulnerable to heat due to thermoregulatory ability diminishing with age (e.g., reduced sweat gland output, reduced skin blood flow and a smaller increase in cardiac output), as well as the increased likelihood of living alone and taking medications [60]. Hence, in the age group ≥ 65 years, the RR of women is higher than the RR of men.

The findings for vulnerable populations can play a guiding role in implementing preventive measures. The government should develop heatwave warning systems and inform the public of upcoming heat waves. Local communities should focus on the vulnerable populations during heat waves and take measures such as reducing outdoor activities and using cooling equipment. Meanwhile, medical institutes should make adequate preparations for the increased demand for care during heat waves.

Water supply service, carbon sequestration service, cooling service and biodiversity can protect against mortality during heat waves, whereas cultural service increase heat-related mortality risks. This result is consistent with many previous studies [25,45,62]. Atmospheric blocking with a moisture deficit can lead to a continuous warm period [23,24]. Greenness regulates the local thermal environment by providing shade, absorbing thermal radiation through transpiration and removing greenhouse gases [25]. Urbanization makes the landscape pattern more fragmented and increased land use diversity leads to reduced vegetation coverage. [63]. Thus, the production of culture service may make heat waves and related health issues more severe. The concept of ESs can be integrated into urban planning as a mitigation measure to regulate the local thermal environment. In urban planning, we should pay attention to the carbon sequestration services of plants, enhancing connectivity between landscapes and increasing vegetation cover.

Surprisingly, this study found that some direct effects of ESs on mortality were opposite to what was expected. For example, the CS and NDVI are significantly negatively correlated with mortality in some groups. The effects may be modified by education level, medical care, air conditioning use and socioeconomic status [64,65,66]. Public services such as medical services and education services are improved in the process of urbanization [67]. However, rapid urbanization will inevitably destroy ecosystems and reduce ESs [68]. It is difficult for public services and ecosystem services to reach a positive synergy [40]. Heat-related mortality risks can be offset by increased public services.

One shortcoming of this study is that data on daily temperature was obtained from weather monitoring stations. Indoor heat exposure was not considered. Due to the limitation of mortality data, the study did not consider the long-term changes of ecosystem services. The study did not consider the lag of heat waves and the modification of air pollutants in the DLNM. Socioeconomic and demographic variables were not considered in this study. During heat waves, individual exposure to heat is affected by many factors, such as work properties and the neighborhood environment. Thus, the air temperature cannot accurately represent individual exposure.

## 5. Conclusions

This study analyzed the relationship among ESs, heat waves and heat-related mortality. The results from this study have practical significance for policy making and urban planning. The results reveal the following: (1) An impact chain exists between ecosystem services, heat waves and mortality risk. (2) The impact of carbon sequestration service on heat waves is the strongest. (3) The elderly, women and people with cardiorespiratory diseases are most at risk from heat waves. (4) ESs such as water supply service, carbon sequestration service, cooling service and biodiversity can reduce the mortality risk by reducing heat waves, especially for the elderly and women. Public health interventions are suggested to focus more on the vulnerable populations such as the elderly, women and people suffering from cardiorespiratory diseases. Ecosystem services could be integrated into urban planning to mitigate heat-related health risks and to help focus on carbon sequestration services.

## Figures and Tables

**Table 1:** Data, resolution and sources.

Data	Resolution	Sources
Precipitation (mm)	1 km × 1 km	National Earth System Science Data Center
Potential evapotranspiration (mm)	1 km × 1 km	
Land cover	300 m × 300 m	European Space Agency
Net primary production (g C/m^2^)	500 m × 500 m	National Aeronautics and Space Administration
NDVI	1 km × 1 km	Resource and Environmental Science and Data Center
Maximum temperature (^o^C)	weather station	China Meteorological Data Service Center
Relative humidity (%)		
Wind speed (m/s)		
Mortality	city	Jiangsu Provincial Center for Disease Prevention and Control

**Table 2:** The results of mediating effects of ESs on mortality risks through HWMI.

	WY	CS	NDVI	COHESION	SHDI
Nonaccidental	-	−0.448	-	−0.362	0.437
Cardiorespiratory	-	−0.585	−0.212	−0.462	0.552
Hypertensive diseases	-	−0.475	−0.125	−0.216	0.400
IHD	-	−0.530	−0.187	-	0.410
Stroke	−0.149	−0.534	−0.227	−0.507	0.528
COPD	−0.134	−0.700	−0.284	−0.512	0.636
Men	-	−0.440	-	−0.436	0.450
Women	-	−0.690	−0.275	−0.475	0.619
15–64	-	-	-	−0.130	0.177
65–74	−0.213	-	−0.154	−0.396	0.294
≥75	-	−0.636	−0.232	−0.453	0.581
15–64 men	-	-	-	−0.146	-
15–64 women	-	−0.320	-	−0.290	0.332
65–74 men	−0.171	−0.369	−0.180	−0.450	0.399
65–74 women	−0.221	-	−0.121	−0.330	-
≥75 men	-	−0.540	−0.185	−0.453	0.521
≥75 women	-	−0.707	−0.279	−0.433	0.615

## Data Availability

All data included in the analysis during this study are available on request from the corresponding author.

## Supplementary Materials

The following supporting information can be downloaded at: https://www.mdpi.com/article/10.3390/ijerph20032750/s1. Figure S1. Ecosystem services of Jiangsu Province; Figure S2. Spatial-temporal patterns of HWMI; Figure S3. Mortality relative risk caused by heat from 2007 to 2015; Table S1. Estimated effects of heat (95% confidence interval) on cause-specific mortality during 2007 to 2015 in 3 cities; Table S2. Estimated effects of heat (95% confidence interval) on cardiorespiratory mortality during 2007 to 2015 in different sexes and age-groups in 3 cities; Table S3. The minimum mortality temperature (MMT) stratified by cause of death; Table S4. The minimum mortality temperature (MMT) of cardiorespiratory diseases stratified by sex and age; Table S5. Sensitivity analysis for the RR of cause-specific mortality caused by extreme heat in three cities; Table S6. Sensitivity analysis for the RR of cardiorespiratory mortality in different sexes and age-groups caused by extreme heat in three cities; Table S7. Ecosystem services values of Jiangsu Province, Nanjing, Suzhou and Yancheng; Table S8. Correlation between ESs and HWMI; Table S9. Correlation between ESs and cause-specific mortality risk associated with heat; Table S10. Correlation between ESs and cardiorespiratory mortality risk in different groups associated with heat; Table S11. Results of paths and effects for different cause-special mortality risk; Table S12. Results of paths and effects for different sexes and age-groups mortality risk; Table S13. Results of Granger causality test between ESs and HWMI.

## References

[1] Wang Q., Zhang Y., Ban J., Zhu H., Xu H., Li T. (2021). The Relationship between Population Heat Vulnerability and Urbanization Levels: A County-Level Modeling Study across China. Environ. Int..

[2] Wang P., Zhang W., Liu J., He P., Wang J., Huang L., Zhang B. (2023). Analysis and Intervention of Heatwave Related Economic Loss: Comprehensive Insights from Supply, Demand, and Public Expenditure into the Relationship between the Influencing Factors. J. Environ. Manag..

[3] McGregor G.R., Bessmoulin P., Ebi K., Menne B. (2015). Heatwaves and Health: Guidance on Warning-System Development.

[4] Luber G., McGeehin M. (2008). Climate Change and Extreme Heat Events. Am. J. Prev. Med..

[5] Perkins-Kirkpatrick S.E., Lewis S.C. (2020). Increasing Trends in Regional Heatwaves. Nat. Commun..

[6] Yin P., Chen R., Wang L., Liu C., Niu Y., Wang W., Jiang Y., Liu Y., Liu J., Qi J. (2018). The Added Effects of Heatwaves on Cause-Specific Mortality: A Nationwide Analysis in 272 Chinese Cities. Environ. Int..

[7] Yang J., Yin P., Sun J., Wang B., Zhou M., Li M., Tong S., Meng B., Guo Y., Liu Q. (2019). Heatwave and Mortality in 31 Major Chinese Cities: Definition, Vulnerability and Implications. Sci. Total Environ..

[8] Saucy A., Ragettli M.S., Vienneau D., de Hoogh K., Tangermann L., Schäffer B., Wunderli J.M., Probst-Hensch N., Röösli M. (2021). The Role of Extreme Temperature in Cause-Specific Acute Cardiovascular Mortality in Switzerland: A Case-Crossover Study. Sci. Total Environ..

[9] Chen K., Horton R.M., Bader D.A., Lesk C., Jiang L., Jones B., Zhou L., Chen X., Bi J., Kinney P.L. (2017). Impact of Climate Change on Heat-Related Mortality in Jiangsu Province, China. Environ. Pollut..

[10] Kollanus V., Tiittanen P., Lanki T. (2021). Mortality Risk Related to Heatwaves in Finland–Factors Affecting Vulnerability. Environ. Res..

[11] Henrique I., Vilella S., Nascimento M., Rodrigues T., Leite W., Cirino G., Ignotti E. (2023). International Journal of Hygiene and Environmental Health Heat Waves and Mortality in the Brazilian Amazon: Effect Modification by Heat Wave Characteristics, Population Subgroup, and Cause of Death. Int. J. Hyg. Environ. Health.

[12] Dimitrova A., Ingole V., Basagaña X., Ranzani O., Milà C., Ballester J., Tonne C. (2021). Association between Ambient Temperature and Heat Waves with Mortality in South Asia: Systematic Review and Meta-Analysis. Environ. Int..

[13] Kovats R.S., Hajat S. (2008). Heat Stress and Public Health: A Critical Review. Annu. Rev. Public Health.

[14] van Steen Y., Ntarladima A.-M., Grobbee R., Karssenberg D., Vaartjes I. (2019). Sex Differences in Mortality after Heat Waves: Are Elderly Women at Higher Risk?. Int. Arch. Occup. Environ. Health.

[15] Bai L., Ding G., Gu S., Bi P., Su B., Qin D., Xu G., Liu Q. (2014). The Effects of Summer Temperature and Heat Waves on Heat-Related Illness in a Coastal City of China, 2011-2013. Environ. Res..

[16] Rocklöv J., Forsberg B., Ebi K., Bellander T. (2014). Susceptibility to Mortality Related to Temperature and Heat and Cold Wave Duration in the Population of Stockholm County, Sweden. Glob. Health Action.

[17] Díaz J., López I.A., Carmona R., Mirón I.J., Luna M.Y., Linares C. (2018). Short-Term Effect of Heat Waves on Hospital Admissions in Madrid: Analysis by Gender and Comparision with Previous Findings. Environ. Pollut..

[18] Dubey A.K., Lal P., Kumar P., Kumar A., Dvornikov A.Y. (2021). Present and Future Projections of Heatwave Hazard-Risk over India: A Regional Earth System Model Assessment. Environ. Res..

[19] Perkins S.E., Alexander L.V., Nairn J.R. (2012). Increasing Frequency, Intensity and Duration of Observed Global Heatwaves and Warm Spells. Geophys. Res. Lett..

[20] Sebastiani A., Marando F., Manes F. (2021). Mismatch of Regulating Ecosystem Services for Sustainable Urban Planning: PM10 Removal and Urban Heat Island Effect Mitigation in the Municipality of Rome (Italy). Urban For. Urban Green..

[21] Ainscough J., de Vries Lentsch A., Metzger M., Rounsevell M., Schröter M., Delbaere B., de Groot R., Staes J. (2019). Navigating Pluralism: Understanding Perceptions of the Ecosystem Services Concept. Ecosyst. Serv..

[22] Millennium Ecosystem Assessment (2005). Ecosystems and Human Well-Being.

[23] Matsueda M. (2011). Predictability of Euro-Russian Blocking in Summer of 2010. Geophys. Res. Lett..

[24] Pfahl S., Wernli H. (2012). Quantifying the Relevance of Atmospheric Blocking for Co-Located Temperature Extremes in the Northern Hemisphere on (Sub-)Daily Time Scales. Geophys. Res. Lett..

[25] Aram F., Higueras García E., Solgi E., Mansournia S. (2019). Urban Green Space Cooling Effect in Cities. Heliyon.

[26] Marando F., Salvatori E., Sebastiani A., Fusaro L., Manes F. (2019). Regulating Ecosystem Services and Green Infrastructure: Assessment of Urban Heat Island Effect Mitigation in the Municipality of Rome, Italy. Ecol. Modell..

[27] Zhang H., Liu L., Zeng Y., Liu M., Bi J., Ji J.S. (2021). Effect of Heatwaves and Greenness on Mortality among Chinese Older Adults. Environ. Pollut..

[28] Burkart K., Meier F., Schneider A., Breitner S., Canário P., Alcoforado M.J., Scherer D., Endlicher W. (2016). Modification of Heat-Related Mortality in an Elderly Urban Population by Vegetation (Urban Green) and Proximity to Water (Urban Blue): Evidence from Lisbon, Portugal. Environ. Health Perspect..

[29] Marvuglia A., Koppelaar R., Rugani B. (2020). The Effect of Green Roofs on the Reduction of Mortality Due to Heatwaves: Results from the Application of a Spatial Microsimulation Model to Four European Cities. Ecol. Modell..

[30] Choi H.M., Lee W., Roye D., Heo S., Urban A., Entezari A., Vicedo-Cabrera A.M., Zanobetti A., Gasparrini A., Analitis A. (2022). Effect Modification of Greenness on the Association between Heat and Mortality: A Multi-City Multi-Country Study. eBioMedicine.

[31] Gronlund C.J., Zanobetti A., Wellenius G.A., Schwartz J.D., O’Neill M.S. (2016). Vulnerability to Renal, Heat and Respiratory Hospitalizations During Extreme Heat among U.S. Elderly. Clim. Chang..

[32] Xu Y., Dadvand P., Barrera-Gómez J., Sartini C., Marí-Dell’Olmo M., Borrell C., Medina-Ramón M., Sunyer J., Basagaña X. (2013). Differences on the Effect of Heat Waves on Mortality by Sociodemographic and Urban Landscape Characteristics. J. Epidemiol. Community Health.

[33] Pu Y., Wang Y., Wang P. (2022). Driving Effects of Urbanization on City-Level Carbon Dioxide Emissions: From Multiple Perspectives of Urbanization. Int. J. Urban Sci..

[34] Wang P., Yu P., Huang L., Zhang Y. (2022). An Integrated Technical, Economic, and Environmental Framework for Evaluating the Rooftop Photovoltaic Potential of Old Residential Buildings. J. Environ. Manag..

[35] Boithias L., Acuña V., Vergoñós L., Ziv G., Marcé R., Sabater S. (2014). Assessment of the Water Supply: DEmand Ratios in a Mediterranean Basin under Different Global Change Scenarios and Mitigation Alternatives. Sci. Total Environ..

[36] Zhang L., Dawes W.R., Walker G.R. (2001). Response of Mean Annual Evapotranspiration to Vegetation Changes at Catchment Scale. Water Resour. Res..

[37] Zhang K., Lyu Y., Fu B., Yin L., Yu D. (2020). The Effects of Vegetation Coverage Changes on Ecosystem Service and Their Threshold in the Loess Plateau. Dili Xuebao/Acta Geogr. Sin..

[38] Lu N., Sun G., Feng X., Fu B. (2013). Water Yield Responses to Climate Change and Variability across the North-South Transect of Eastern China (NSTEC). J. Hydrol..

[39] Cao W., Li R., Chi X., Chen N., Chen J., Zhang H., Zhang F. (2017). Island Urbanization and Its Ecological Consequences: A Case Study in the Zhoushan Island, East China. Ecol. Indic..

[40] Hou X., Wu S., Chen D., Cheng M., Yu X., Yan D., Dang Y., Peng M. (2021). Can Urban Public Services and Ecosystem Services Achieve Positive Synergies?. Ecol. Indic..

[41] Bastin J.F., Finegold Y., Garcia C., Mollicone D., Rezende M., Routh D., Zohner C.M., Crowther T.W. (2019). The Global Tree Restoration Potential. Science.

[42] Li B., Chen D., Wu S., Zhou S., Wang T., Chen H. (2016). Spatio-Temporal Assessment of Urbanization Impacts on Ecosystem Services: Case Study of Nanjing City, China. Ecol. Indic..

[43] Antrop M. (2005). Why Landscapes of the Past Are Important for the Future. Landsc. Urban Plan..

[44] Uuemaa E., Mander Ü., Marja R. (2013). Trends in the Use of Landscape Spatial Metrics as Landscape Indicators: A Review. Ecol. Indic..

[45] Chen A., Yao L., Sun R., Chen L. (2014). How Many Metrics Are Required to Identify the Effects of the Landscape Pattern on Land Surface Temperature?. Ecol. Indic..

[46] Bing Z., Qiu Y., Huang H., Chen T., Zhong W., Jiang H. (2021). Spatial Distribution of Cultural Ecosystem Services Demand and Supply in Urban and Suburban Areas: A Case Study from Shanghai, China. Ecol. Indic..

[47] Russo S., Dosio A., Graversen R.G., Sillmann J., Carrao H., Dunbar M.B., Singleton A., Montagna P., Barbola P., Vogt J.V. (2014). Magnitude of Extreme Heat Waves in Present Climate and Their Projection in a Warming World. J. Geophys. Res. Atmos..

[48] Ma Y., Zhou L., Chen K. (2020). Burden of Cause-Specific Mortality Attributable to Heat and Cold: A Multicity Time-Series Study in Jiangsu Province, China. Environ. Int..

[49] Gasparrini A., Guo Y., Hashizume M., Lavigne E., Zanobetti A., Schwartz J., Tobias A., Tong S., Rocklöv J., Forsberg B. (2015). Mortality Risk Attributable to High and Low Ambient Temperature: A Multicountry Observational Study. Lancet.

[50] Gasparrini A. (2011). Distributed Lag Linear and Non-Linear Models in R: The Package Dlnm. J. Stat. Softw..

[51] Chen R., Yin P., Wang L., Liu C., Niu Y., Wang W., Jiang Y., Liu Y., Liu J., Qi J. (2018). Association between Ambient Temperature and Mortality Risk and Burden: Time Series Study in 272 Main Chinese Cities. BMJ.

[52] Wu W., Xiao Y., Li G., Zeng W., Lin H., Rutherford S., Xu Y., Luo Y., Xu X., Chu C. (2013). Temperature–Mortality Relationship in Four Subtropical Chinese Cities: A Time-Series Study Using a Distributed Lag Non-Linear Model. Sci. Total Environ..

[53] Wang C., Sheng Y., Wang J., Wang Y., Wang P. (2022). Air Pollution and Human Health: Investigating the Moderating Effect of the Built Environment. Remote Sens..

[54] Wang P., Yu P., Lu J., Zhang Y. (2022). The Mediation Effect of Land Surface Temperature in the Relationship between Land Use-Cover Change and Energy Consumption under Seasonal Variations. J. Clean. Prod..

[55] Hayes A.F. (2017). Introduction to Mediation, Moderation, and Conditional Process Analysis: A Regression-Based Approach.

[56] Wang P., Zhu Y., Yu P. (2022). Assessment of Urban Flood Vulnerability Using the Integrated Framework and Process Analysis: A Case from Nanjing, China. Int. J. Environ. Res. Public Health.

[57] Shen X., Ma L.J.C. (2005). Privatization of Rural Industry and de Facto Urbanization from below in Southern Jiangsu, China. Geoforum.

[58] Liu Y., Wang Z., Liu X., Zhang B. (2021). Complexity of the Relationship between 2D/3D Urban Morphology and the Land Surface Temperature: A Multiscale Perspective. Environ. Sci. Pollut. Res..

[59] Wang P., Zhang Y., Wang J., Wang Y., Huang L. (2022). Projected Attributable Mortality of Characteristic Populations Related to Different Definitions of Heat: Evidence from Jiangsu Province, China. Urban Clim..

[60] Cheng J., Xu Z., Bambrick H., Su H., Tong S., Hu W. (2018). Heatwave and Elderly Mortality: An Evaluation of Death Burden and Health Costs Considering Short-Term Mortality Displacement. Environ. Int..

[61] Kim E.J., Kim H. (2017). Effect Modification of Individual- and Regional-Scale Characteristics on Heat Wave-Related Mortality Rates between 2009 and 2012 in Seoul, South Korea. Sci. Total Environ..

[62] Lo S.H., Chen C.T., Russo S., Huang W.R., Shih M.F. (2021). Tracking Heatwave Extremes from an Event Perspective. Weather Clim. Extrem..

[63] Peng J., Jia J., Liu Y., Li H., Wu J. (2018). Seasonal Contrast of the Dominant Factors for Spatial Distribution of Land Surface Temperature in Urban Areas. Remote Sens. Environ..

[64] Ma W., Chen R., Kan H. (2014). Temperature-Related Mortality in 17 Large Chinese Cities: How Heat and Cold Affect Mortality in China. Environ. Res..

[65] Liu T., Ren Z., Zhang Y., Feng B., Lin H., Xiao J., Zeng W., Li X., Li Z., Rutherford S. (2019). Modification Effects of Population Expansion, Ageing, and Adaptation on Heat-Related Mortality Risks under Different Climate Change Scenarios in Guangzhou, China. Int. J. Environ. Res. Public Health.

[66] Lin J., Leung J., Yu B., Woo J., Kwok T., Ka-Lun Lau K. (2021). Socioeconomic Status as an Effect Modifier of the Association between Built Environment and Mortality in Elderly Hong Kong Chinese: A Latent Profile Analysis. Environ. Res..

[67] Yin C., Shao C., Dong C., Wang X. (2019). Happiness in Urbanizing China: The Role of Commuting and Multi-Scale Built Environment across Urban Regions. Transp. Res. Part D Transp. Environ..

[68] Wu S., Zhou S., Bao H., Chen D., Wang C., Li B., Tong G., Yuan Y., Xu B. (2019). Improving Risk Management by Using the Spatial Interaction Relationship of Heavy Metals and PAHs in Urban Soil. J. Hazard. Mater..

