# Supplementary material for: Mediating Effect of Heat Waves between Ecosystem Services and Heat-Related Mortality of Characteristic Populations: Evidence from Jiangsu Province, China
Source: Int J Environ Res Public Health. 2023 Feb 3;20(3):2750. doi: 10.3390/ijerph20032750 (PMC9915879; doi:10.3390/ijerph20032750)
Supplement: Supplementary file 1 [file ijerph-20-02750-s001.zip › ijerph-2141955-supplementary.pdf]

Supplementary materials

Title: Mediating effect of heat wave between ecosystem services and heat-related mortality of characteristic populations

Table of Contents

Figure S1. Ecosystem services of Jiangsu Province.

Figure S2. Spatial-temporal patterns of HWMI.

Figure S3. Mortality relative risk caused by heat from 2007 to 2015

Table S1. Estimated effects of heat (95% confidence interval) on cause-specific mortality during 2007 to 2015 in 3 cities.

Table S2. Estimated effects of heat (95% confidence interval) on cardiorespiratory mortality during 2007 to 2015 in different sexes and age-groups in 3 cities.

Table S3. The minimum mortality temperature (MMT) stratified by cause of death.

Table S4. The minimum mortality temperature (MMT) of cardiorespiratory diseases stratified by sex and age.

Table S5. Sensitivity analysis for the RR of cause-specific mortality caused by extreme heat in three cities.

Table S6. Sensitivity analysis for the RR of cardiorespiratory mortality in different sexes and age-groups caused by extreme heat in three cities.

Table S7. Ecosystem services values of Jiangsu Province, Nanjing, Suzhou and

Yancheng.

Table S8. Correlation between ESs and HWMI.

Table S9. Correlation between ESs and cause-specific mortality risk associated with heat.

Table S10. Correlation between ESs and cardiorespiratory mortality risk in different groups associated with heat

Table S11. Results of paths and effects for different cause-special mortality risk.

Table S12. Results of paths and effects for different sexes and age-groups mortality risk.

Table S13. Results of Granger causality test between ESs and HWMI.

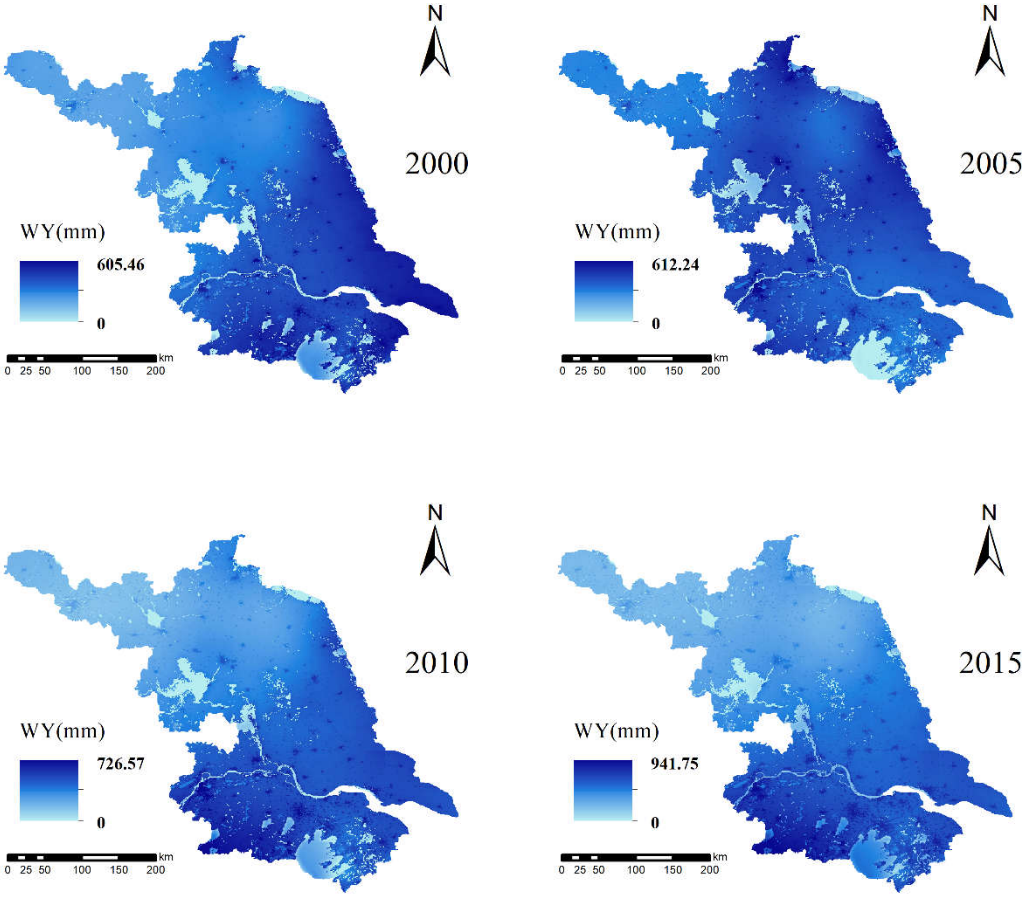

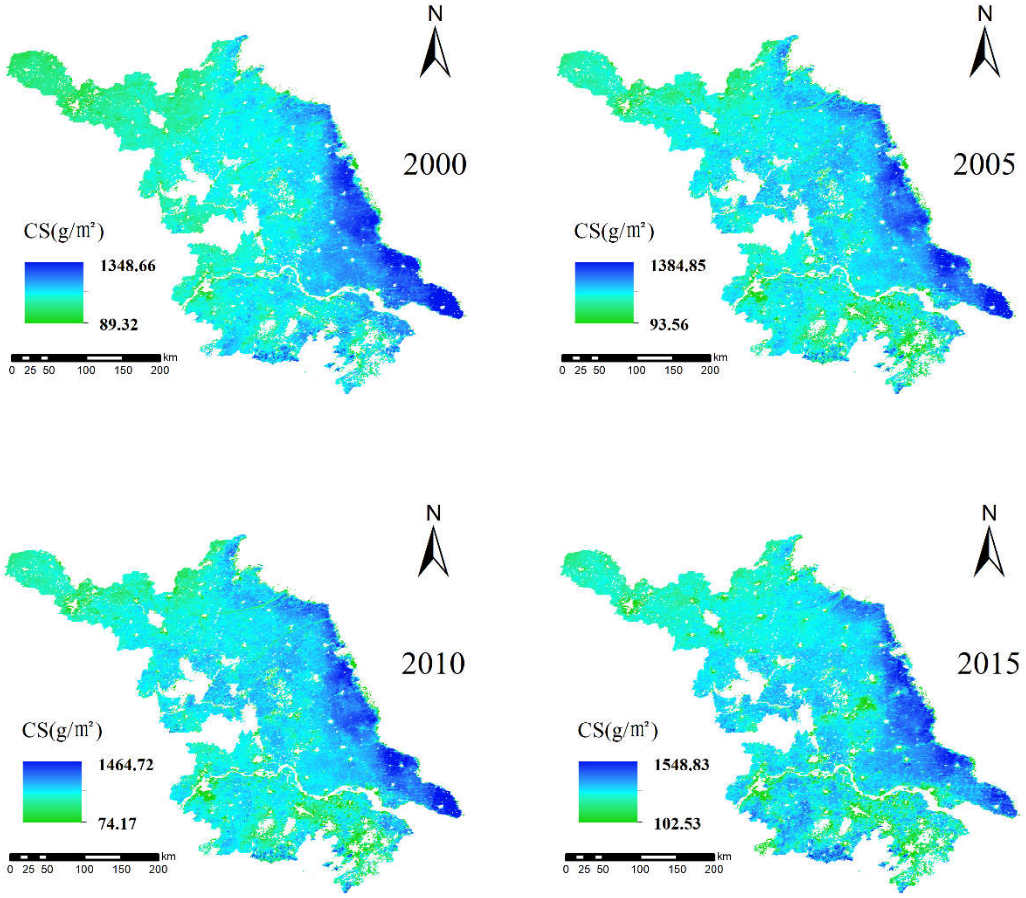

N
2000
CS(g/m²)
1348.66
89.32
0 25 50 100 150 200 km
N
2005
CS(g/m²)
1384.85
93.56
0 25 50 100 150 200 km
N
2010
CS(g/m²)
1464.72
74.17
0 25 50 100 150 200 km
N
2015
CS(g/m²)
1548.83
102.53
0 25 50 100 150 200 km

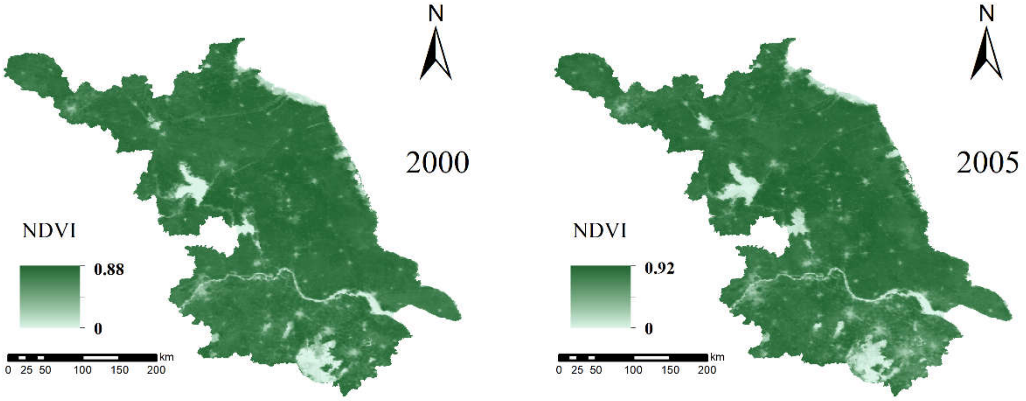

N
2000
NDVI
0.88
0
0 25 50 100 150 200 km
N
2005
NDVI
0.92
0
0 25 50 100 150 200 km

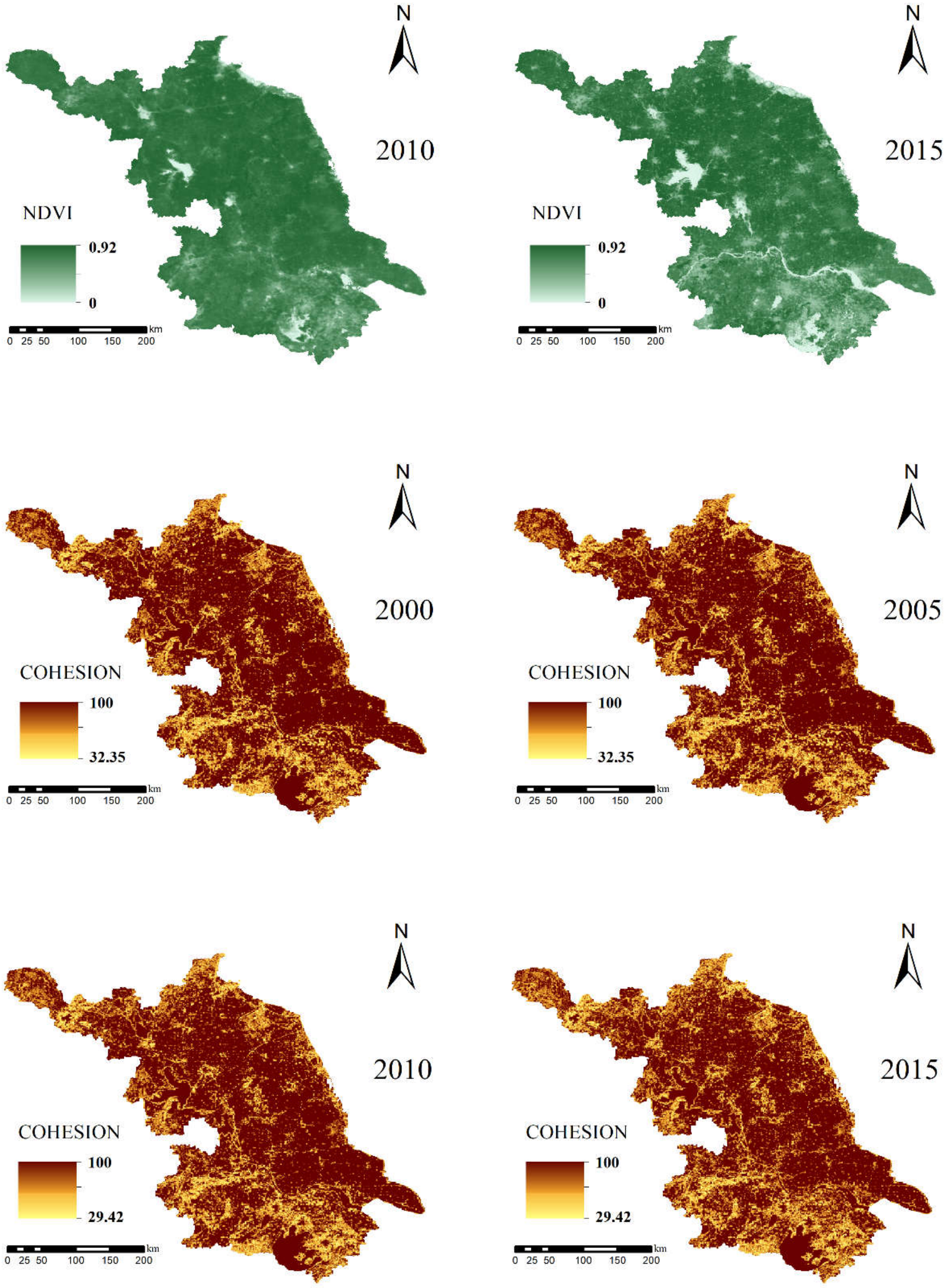
N
2010
NDVI
0.92
0
km
0 25 50 100 150 200
N
2015
NDVI
0.92
0
km
0 25 50 100 150 200
N
2000
COHESION
100
32.35
km
0 25 50 100 150 200
N
2005
COHESION
100
32.35
km
0 25 50 100 150 200
N
2010
COHESION
100
29.42
km
0 25 50 100 150 200
N
2015
COHESION
100
29.42
km
0 25 50 100 150 200

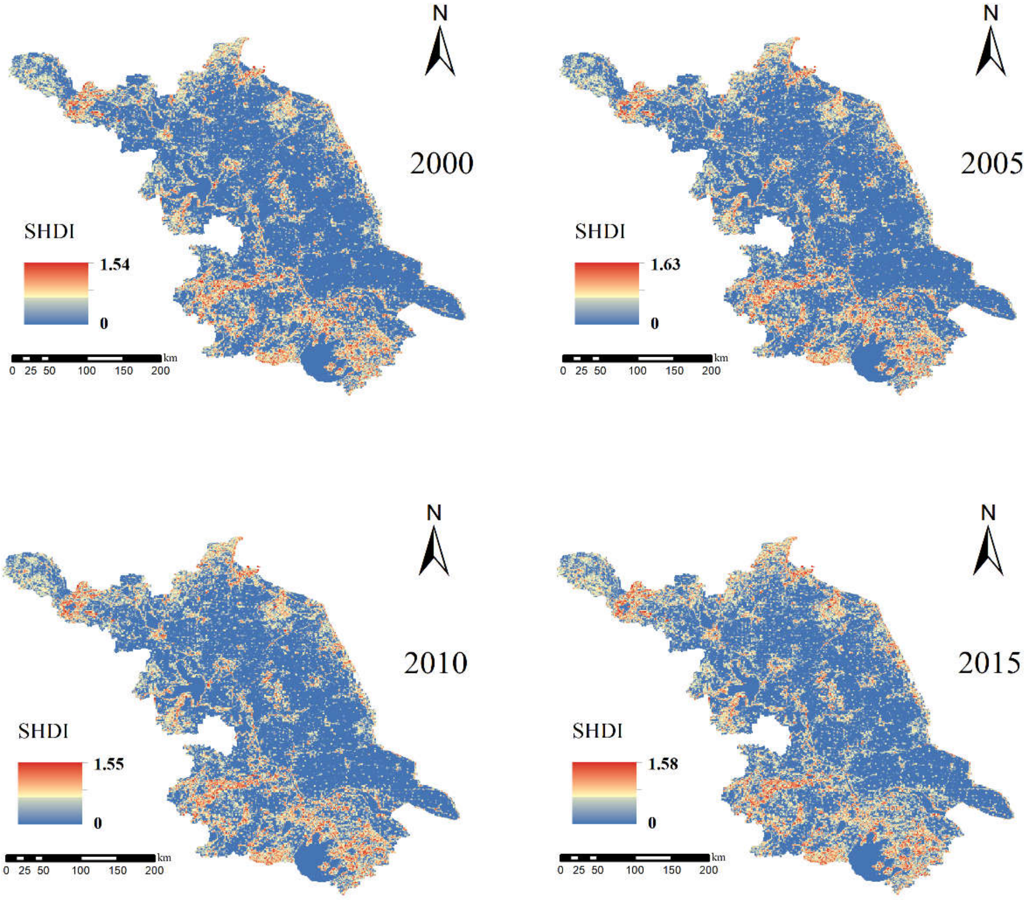

Figure S1. Ecosystem services of Jiangsu Province.

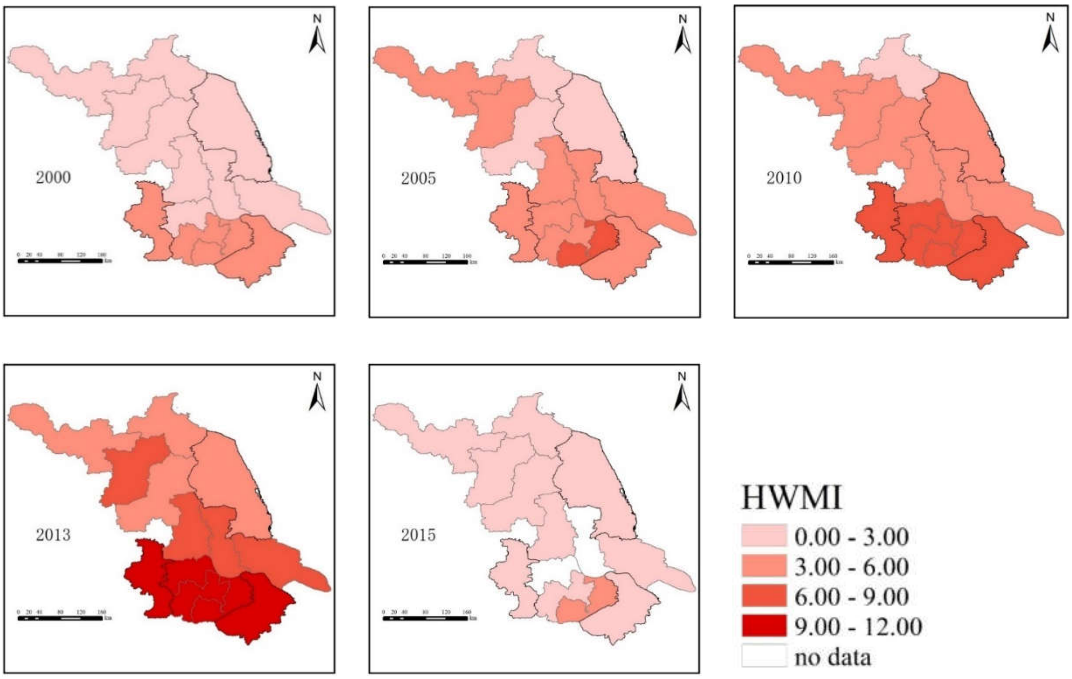

Figure S2. Spatial-temporal patterns of HWMI.

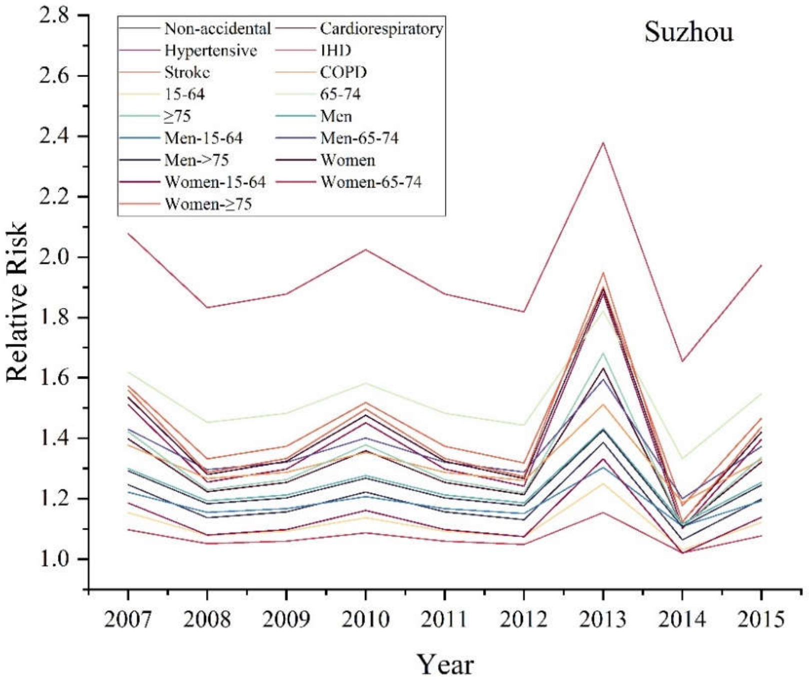

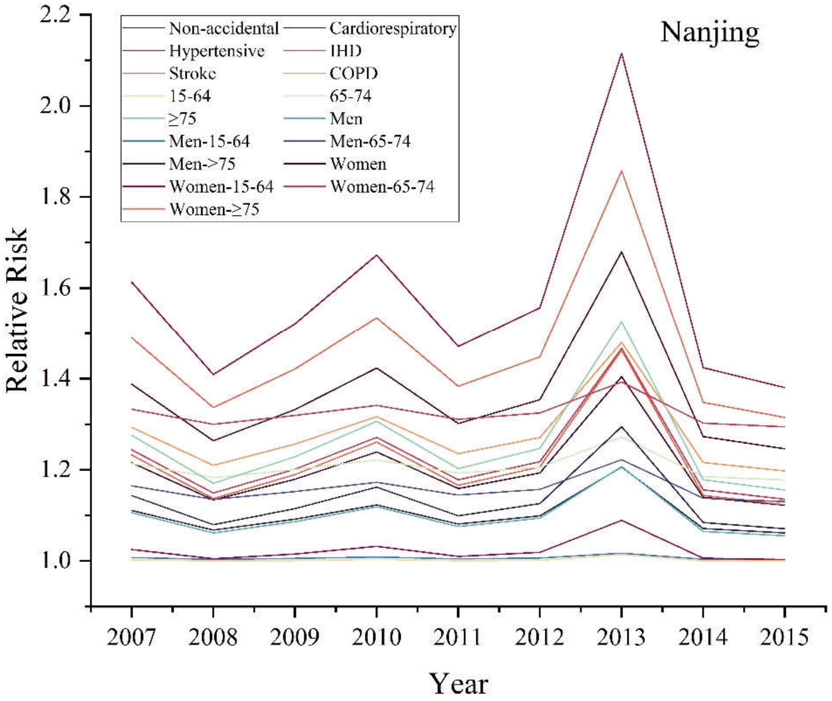

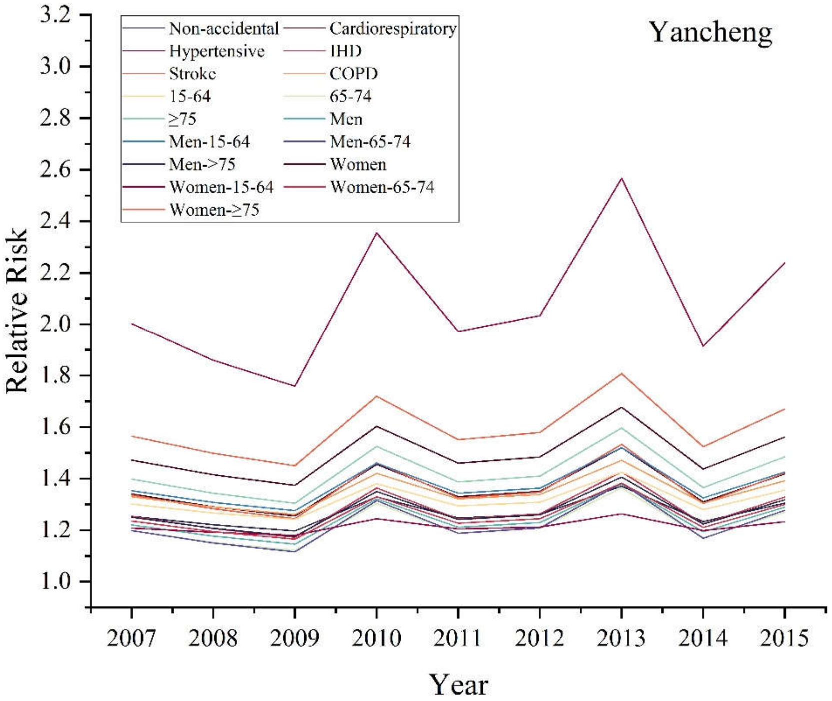

Figure S3. Mortality relative risk caused by heat from 2007 to 2015.

Table S1. Estimated effects of heat (95% confidence interval) on cause-specific mortality during 2007 to 2015 in 3 cities.

Cause of death	Nanjing	Suzhou	Yancheng
	RR	RR	RR
Non-accidental	1.10(1.04,1.15)	1.15(1.08,1.22)	1.27(1.18,1.36)
Cardiorespiratory diseases	1.19(1.12,1.26)	1.28(1.21,1.35)	1.36(1.25,1.49)
Hypertensive diseases	1.54(1.16,2.03)	1.33(1.16,1.53)	2.07(1.27,3.37)
IHD	1.21(1.09,1.34)	1.06(0.93,1.20)	1.27(1.08,1.50)
Stroke	1.20(1.11,1.29)	1.40(1.26,1.56)	1.36(1.21,1.53)
COPD	1.26(1.03,1.55)	1.30(1.09,1.55)	1.35(1.16,1.56)

IHD: ischeamic heart diseases, COPD: chronic obstructive pulmonary disease

Table S2. Estimated effects of heat (95% confidence interval) on cardiorespiratory mortality during 2007 to 2015 in different sexes and age-groups in 3 cities.

sex, age	Nanjing	Suzhou	Yancheng
	RR	RR	RR
**All**			
All ages	1.19(1.12,1.26)	1.28(1.21,1.35)	1.36(1.25,1.49)
15-64	1.01(0.93,1.09)	1.10(0.95,1.27)	1.32(1.07,1.61)
65-74	1.20(1.02,1.42)	1.50(1.24,1.82)	1.22(1.09,1.36)
≥75	1.24(1.16,1.32)	1.29(1.21,1.36)	1.42(1.28,1.58)
**Men**			
All ages	1.09(1.02,1.17)	1.23(1.11,1.35)	1.24(1.11,1.38)
15-64	1.01(0.89,1.13)	1.18(0.87,1.59)	1.37(1.06,1.77)

65-74	1.15(0.95,1.40)	1.34(1.04,1.68)	1.22(1.06,1.41)
≥75	1.12(1.03,1.21)	1.22(1.09,1.36)	1.27(1.11,1.44)
**Women**			
All ages	1.34(1.20,1.50)	1.35(1.27,1.45)	1.50(1.33,1.68)
15-64	1.02(0.90,1.14)	1.11(0.89,1.39)	1.21(0.87,1.70)
65-74	1.32(1.01,1.74)	1.91(1.36,2.68)	1.25(0.99,1.59)
≥75	1.43(1.27,1.62)	1.37(1.27,1.46)	1.59(1.40,1.81)

Table S3. The minimum mortality temperature (MMT) stratified by cause of death.

Cause of death	Nanjing	Suzhou	Yancheng
	Temperature (°C)	Temperature (°C)	Temperature (°C)
Non-accidental	30.64	30.56	14.41
Cardiorespiratory diseases	29.88	29.87	13.61
Hypertensive diseases	25.76	31.39	10.33
IHD	30.2	31.56	14.89
Stroke	30.91	22.62	13.9
COPD	19.55	19.13	13.17

IHD: ischeamic heart diseases, COPD: chronic obstructive pulmonary disease

Table S4. The minimum mortality temperature (MMT) of cardiorespiratory diseases stratified by sex and age.

sex, age	Nanjing	Suzhou	Yancheng
	Temperature (°C)	Temperature (°C)	Temperature (°C)
**All**			
All ages	29.88	29.87	13.61
15-64	35.29	31.74	11.31
65-74	20.7	17.76	29.95
≥75	29.75	30.41	13.98
**Men**			
All ages	31.38	23.34	15.37
15-64	33.08	15.88	11.21
65-74	23.73	21.02	30.38
≥75	31.76	24.97	18.54
**Women**			
All ages	19.45	30.75	12.39
15-64	34.39	32.84	11.52
65-74	15.71	13.45	16.04
≥75	18.92	30.98	12.07

Table S5. Sensitivity analysis for the RR of cause-specific mortality caused by extreme heat in three cities.

	Adjustment to model	Nanjing	Suzhou	Yancheng

Non-accidental	Main model	1.10(1.04,1.15)	1.15(1.08,1.22)	1.27(1.18,1.36)
	Time: 4 df/year	1.15(1.08,1.21)	1.20(1.15,1.25)	1.34(1.26,1.42)
	Time: 6 df/year	1.11(1.06,1.17)	1.17(1.13,1.22)	1.26(1.18,1.35)
	Lag: 4 days	1.12(1.06,1.19)	1.21(1.14,1.29)	1.29(1.21,1.38)
	Lag: 8 days	1.08(1.03,1.14)	1.16(1.12,1.21)	1.25(1.17,1.35)
Cardiorespiratory deaths	Main model	1.19(1.12,1.26)	1.28(1.21,1.35)	1.36(1.25,1.49)
	Time: 4 df/year	1.24(1.17,1.31)	1.36(1.27,1.44)	1.51(1.40,1.62)
	Time: 6 df/year	1.21(1.14,1.28)	1.27(1.21,1.34)	1.34(1.23,1.47)
	Lag: 4 days	1.22(1.13,1.33)	1.35(1.24,1.46)	1.39(1.28,1.51)
	Lag: 8 days	1.17(1.10,1.24)	1.26(1.19,1.33)	1.36(1.24,1.49)
Hypertensive diseases	Main model	1.54(1.16,2.03)	1.33(1.16,1.53)	2.07(1.27,3.37)
	Time: 4 df/year	1.74(1.36,2.23)	1.40(1.23,1.60)	1.78(1.16,2.75)
	Time: 6 df/year	1.52(1.15,2.00)	1.33(1.17,1.52)	2.09(1.27,3.45)
	Lag: 4 days	1.60(1.22,2.09)	1.40(1.09,1.78)	2.07(1.33,3.22)
	Lag: 8 days	1.46(1.10.1.94)	1.33(1.15,1.54)	1.98(1.17,3.34)
Ischaemic heart diseases	Main model	1.21(1.09,1.34)	1.06(0.93,1.2)	1.27(1.08,1.50)
	Time: 4 df/year	1.27(1.13,1.42)	1.15(0.99,1.33)	1.44(1.25,1.64)
	Time: 6 df/year	1.23(1.11,1.37)	1.07(0.95,1.20)	1.25(1.12,1.40)
	Lag: 4 days	1.20(1.09,1.32)	1.09(0.96,1.24)	1.30(1.11,1.51)
	Lag: 8 days	1.18(1.07,1.31)	1.01(0.88,1.17)	1.26(1.06,1.51)
Stroke	Main model	1.2(1.11,1.29)	1.40(1.26,1.56)	1.36(1.21,1.53)

	Time: 4 df/year	1.22(1.13,1.31)	1.33(1.22,1.45)	1.37(1.24,1.51)
	Time: 6 df/year	1.22(1.13,1.31)	1.38(1.26,1.51)	1.34(1.19,1.51)
	Lag: 4 days	1.21(1.13,1.30)	1.49(1.33,1.65)	1.40(1.26,1.56)
	Lag: 8 days	1.18(1.10,1.28)	1.40(1.27,1.54)	1.37(1.21,1.54)
Chronic obstructive pulmonary disease	Main model	1.26(1.03,1.55)	1.30(1.09,1.55)	1.35(1.16,1.56)
	Time: 4 df/year	1.52(1.28,1.80)	1.61(1.40,1.86)	1.73(1.53,1.95)
	Time: 6 df/year	1.28(1.05,1.56)	1.22(1.05,1.42)	1.33(1.15,1.54)
	Lag: 4 days	1.36(1.12,1.65)	1.37(1.15,1.63)	1.37(1.19,1.57)
	Lag: 8 days	1.24(1.01,1.51)	1.24(1.03,1.49)	1.36(1.16,1.58)

Table S6. Sensitivity analysis for the RR of cardiorespiratory mortality in different sexes and age-groups caused by extreme heat in three cities.

	Adjustment to model	Nanjing	Suzhou	Yancheng
**All**				
All ages	Main model	1.19(1.12,1.26)	1.28(1.21,1.35)	1.36(1.25,1.49)
	Time:4 df/year	1.24(1.17,1.31)	1.36(1.27,1.44)	1.51(1.40,1.62)
	Time: 6 df/year	1.21(1.14,1.28)	1.27(1.21,1.34)	1.34(1.23,1.47)
	Lag: 4 days	1.22(1.13,1.33)	1.35(1.24,1.46)	1.39(1.28,1.51)
	Lag: 8 days	1.17(1.10,1.24)	1.26(1.19,1.33)	1.36(1.24,1.49)
15-64	Main model	1.01(0.93,1.09)	1.10(0.95,1.27)	1.32(1.07,1.61)
	Time: 4 df/year	1.03(0.93,1.15)	1.17(0.95,1.43)	1.35(1.13,1.61)
	Time: 6 df/year	1.01(0.93,1.09)	1.12(0.98,1.28)	1.28(1.04,1.57)
	Lag: 4 days	1.00(0.96,1.04)	1.11(0.97,1.27)	1.30(1.08,1.57)

	Lag: 8 days	1.00(0.98,1.02)	1.11(0.95,1.29)	1.37(1.10,1.71)
65-74	Main model	1.20(1.02,1.42)	1.50(1.24,1.82)	1.22(1.09,1.36)
	Time: 4 df/year	1.34(1.17,1.55)	1.45(1.23,1.70)	1.29(1.14,1.46)
	Time: 6 df/year	1.22(1.04,1.44)	1.43(1.18,1.73)	1.24(1.11,1.38)
	Lag: 4 days	1.25(1.07,1.46)	1.56(1.30,1.87)	1.24(1.11,1.38)
	Lag: 8 days	1.13(0.96,1.32)	1.50(1.22,1.84)	1.20(1.07,1.35)
≥75	Main model	1.24(1.16,1.32)	1.29(1.21,1.36)	1.42(1.28,1.58)
	Time: 4 df/year	1.34(1.24,1.45)	1.36(1.28,1.45)	1.61(1.48,1.75)
	Time: 6 df/year	1.26(1.17,1.35)	1.29(1.22,1.36)	1.40(1.26,1.55)
	Lag: 4 days	1.28(1.17,1.40)	1.34(1.22,1.46)	1.46(1.33,1.60)
	Lag: 8 days	1.22(1.14,1.30)	1.27(1.19,1.35)	1.42(1.27,1.58)
**Men**	Main model			
All ages	Time: 4 df/year	1.09(1.02,1.17)	1.23(1.11,1.35)	1.24(1.11,1.38)
	Time: 6 df/year	1.18(1.08,1.27)	1.31(1.21,1.42)	1.39(1.27,1.52)
	Lag: 4 days	1.11(1.04,1.20)	1.20(1.12,1.29)	1.24(1.15,1.34)
	Lag: 8 days	1.11(1.03,1.20)	1.25(1.13,1.38)	1.27(1.15,1.47)
	Main model	1.08(1.00,1.16)	1.21(1.09,1.33)	1.25(1.11,1.40)
15-64	Time: 4 df/year	1.01(0.89,1.13)	1.18(0.87,1.59)	1.37(1.06,1.77)
	Time: 6 df/year	1.03(0.89,1.19)	1.25(0.96,1.63)	1.42(1.14,1.78)
	Lag: 4 days	1.01(0.90,1.14)	1.17(0.88,1.57)	1.35(1.04,1.74)
	Lag: 8 days	1.01(0.90,1.14)	1.15(0.88,1.51)	1.35(1.07,1.71)
	Main model	1.03(0.76,1.41)	1.15,0.84,1.57)	1.38(1.05,1.82)
65-74	Time: 4 df/year	1.15(0.95,1.40)	1.34(1.04,1.68)	1.22(1.06,1.41)
	Time: 6 df/year	1.28(1.08,1.53)	1.31(1.09,1.58)	1.25(1.08,1.44)
	Lag: 4 days	1.19(0.98,1.45)	1.24(1.01,1.53)	1.24(1.08,1.42)
	Lag: 8 days	1.20(1.00,1.44)	1.40(1.12,1.74)	1.23(1.07,1.40)
	Main model	1.16(0.95,1.41)	1.38(1.08,1.76)	1.20(1.03,1.39)
≥75	Time: 4 df/year	1.12(1.03,1.21)	1.22(1.09,1.36)	1.27(1.11,1.44)
	Time: 6 df/year	1.20(1.09,1.31)	1.27(1.19,1.37)	1.47(1.32,1.63)

	Lag: 4 days	1.14(1.03,1.24)	1.20(1.11,1.30)	1.26(1.14,1.38)
	Lag: 8 days	1.13(1.05,1.23)	1.24(1.11,1.39)	1.31(1.16,1.48)
	Main model	1.10(1.02,1.20)	1.19(1.07,1.32)	1.29(1.13,1.48)
Women	Time: 4 df/year			
All ages	Time: 6 df/year	1.34(1.2,1.5)	1.35(1.27,1.45)	1.50(1.33,1.68)
	Lag: 4 days	1.43(1.30,1.57)	1.40(1.31,1.50)	1.64(1.49,1.80)
	Lag: 8 days	1.35(1.21,1.50)	1.35(1.27,1.44)	1.46(1.30,1.64)
	Main model	1.40(1.26,1.56)	1.45(1.31,1.62)	1.52(1.36,1.69)
	Time: 4 df/year	1.27(1.17,1.37)	1.34(1.24,1.43)	1.48(1.31,1.67)
15-64	Time: 6 df/year	1.02(0.9,1.14)	1.11(0.89,1.39)	1.21(0.87,1.70)
	Lag: 4 days	1.08(0.91,1.28)	1.13(0.90,1.42)	1.22(0.93,1.61)
	Lag: 8 days	1.04(0.89,1.21)	1.11(0.91,1.37)	1.17(0.83,1.65)
	Main model	1.00(0.98,1.02)	1.14(0.91,1.41)	1.22(0.89,1.66)
	Time: 4 df/year	1.04(0.89,1.21)	1.06(0.84,1.34)	1.37(0.97,1.92)
65-74	Time: 6 df/year	1.32(1.01,1.74)	1.91(1.36,2.68)	1.25(0.99,1.59)
	Lag: 4 days	1.46(1.16,1.83)	1.79(1.32,2.42)	1.39(1.15,1.67)
	Lag: 8 days	1.31(0.99,1.73)	1.90(1.35,2.67)	1.23(1.05,1.45)
	Main model	1.38(1.07,1.79)	1.91(1.39,2.62)	1.27(1.01,1.59)
	Time: 4 df/year	1.09(0.83,1.44)	1.78(1.24,2.56)	1.24(0.96,1.60)
≥75	Time: 6 df/year	1.43(1.27,1.62)	1.37(1.27,1.46)	1.59(1.4,1.81)
	Lag: 4 days	1.49(1.35,1.65)	1.41(1.32,1.51)	1.76(1.58,1.96)
	Lag: 8 days	1.44(1.27,1.62)	1.37(1.28,1.46)	1.57(1.38,1.79)
	Main model	1.50(1.34,1.69)	1.44(1.28,1.61)	1.62(1.43,1.82)
	Time: 4 df/year	1.36(1.20,1.53)	1.34(1.25,1.45)	1.55(1.35,1.78)

Table S7. Ecosystem services values of Jiangsu Province, Nanjing, Suzhou and Yancheng.

		WY (mm)	CS (g/m²)	NDVI	COHESION (%)	SHDI
Jiangsu Province	2000	308.717	743.932	0.706	99.623	1.059
	2005	325.94	739.856	0.730	99.618	1.071
	2010	323.344	798.863	0.747	99.617	1.101
	2015	393.316	851.185	0.683	99.608	1.136
Nanjing	2000	314.823	674.331	0.677	98.086	1.406
	2005	370.865	683.621	0.711	98.077	1.412
	2010	463.930	731.055	0.715	98.144	1.414
	2015	596.389	810.272	0.618	98.201	1.424
Suzhou	2000	342.839	764.469	0.576	98.799	1.459
	2005	223.798	659.171	0.530	98.769	1.471
	2010	338.285	647.436	0.565	98.703	1.497
	2015	482.903	733.011	0.455	98.464	1.500
Yancheng	2000	353.622	845.644	0.748	99.500	0.805
	2005	373.877	832.114	0.787	99.494	0.815
	2010	339.413	922.905	0.806	99.486	0.839
	2015	351.647	966.752	0.756	99.481	0.868

Table S8. Correlation between ESs and HWMI.

	WY	CS	NDVI	COHESION	SHDI
HWMI	-0.393 *	-0.658 **	-0.394 *	-0.526 **	0.599 **

* $p<0.05$; ** $p<0.01$

Table S9. Correlation between ESs and cause-specific mortality risk associated with heat.

	WY	CS	NDVI	COHESION	SHDI
Non-accidental	-0.576 **	0.400 *	0.346	0.711 **	-0.577 **
Cardiorespiratory	-0.613 **	0.206	0.216	0.506 **	-0.372
Hypertensive diseases	-0.393 *	0.684 **	0.786 **	0.641 **	-0.783 **
IHD	-0.246	0.556 **	0.787 **	0.308	-0.582 **
Stroke	-0.596 **	-0.141	-0.173	0.271	-0.020
COPD	-0.602 **	0.118	0.211	0.339	-0.260

* p<0.05; ** p<0.01

Table S10. Correlation between ESs and cardiorespiratory mortality risk in different groups associated with heat.

	WY	CS	NDVI	COHESION	SHDI
**All**					
All ages	-0.613**	0.206	0.216	0.506**	-0.372
15-64	-0.412*	0.676**	0.531**	0.932**	-0.836**
65-74	-0.379	-0.598**	-0.698**	-0.119	0.488**
≥75	-0.596**	0.281	0.342	0.493**	-0.433*
**Men**					
All ages	-0.600**	0.089	-0.006	0.530**	-0.271
15-64	-0.427*	0.596**	0.409*	0.924**	-0.765**
65-74	-0.498**	-0.374	-0.452*	0.105	0.228
≥75	-0.617**	0.207	0.172	0.559**	-0.382*
**Women**					
All ages	-0.576**	0.187	0.305	0.345	-0.321
15-64	-0.533**	0.447*	0.321	0.792**	-0.625**

65-74	-0.276	-0.705**	-0.823**	-0.246	0.622**
≥75	-0.525**	0.291	0.459*	0.336	-0.401*

* p<0.05; ** p<0.01

Table S11. Results of paths and effects for different cause-special mortality risk.

X=>M=>Y			Effect of X on M	Direct effect of M on Y
X	M	Y	a	b
WY		Stroke	-0.393*	0.378*
		COPD	-0.393*	0.342*
CS		Non-accidental	-0.658**	0.681**
		Cardiorespiratory	-0.658**	0.889**
		Hypertensive diseases	-0.658**	0.722**
		IHD	-0.658**	0.805**
		Stroke	-0.658**	0.812**
		COPD	-0.658**	1.064**
NDVI	HWMI	Cardiorespiratory	-0.394*	0.537**
		Hypertensive diseases	-0.394*	0.317*
		IHD	-0.394*	0.474**
		Stroke	-0.394*	0.575**
		COPD	-0.394*	0.720**
COHESION		Non-accidental	-0.526**	0.688**
		Cardiorespiratory	-0.526**	0.878*
		Hypertensive diseases	-0.526**	0.410**
		Stroke	-0.526**	0.963**

	COPD	-0.526**	0.974**
	Nonaccidental	0.599**	0.730**
	Cardiorespiratory	0.599**	0.922**
SHDI	Hypertensive diseases	0.599**	0.667**
	IHD	0.599**	0.684**
	Stroke	0.599**	0.882**
	COPD	0.599**	1.062**

* p<0.05** p<0.01

Table S12. Results of paths and effects for different sexes and age-groups mortality risk.

X=>M=>Y			Effect of X on M	Direct effect of M on Y
X	M	Y	a	b
		65-74	-0.393*	0.541**
WY		65-74-men	-0.393*	0.436*
		65-74-women	-0.393*	0.561**
		Men	-0.658**	0.668**
		Women	-0.658**	1.048**
		≥75	-0.658**	0.967**
CS	HWMI	15-64-women	-0.658**	0.486*
		65-74-men	-0.658**	0.561*
		≥75-men	-0.658**	0.821**
		≥75-women	-0.658**	1.075**
		Women	-0.394*	0.699**
NDVI		65-74	-0.394*	0.392**
		≥75	-0.394*	0.589**

	65-74-men	-0.394*	0.457*
	65-74-women	-0.394*	0.306*
	≥75-men	-0.394*	0.469*
	≥75-women	-0.394*	0.709**
	Men	-0.526**	0.829**
	Women	-0.526**	0.903**
	15-64	-0.526**	0.247**
	65-74	-0.526**	0.752**
	≥75	-0.526**	0.862**
COHESION	15-64-men	-0.526**	0.278**
	15-64-women	-0.526**	0.551**
	65-74-men	-0.526**	0.856**
	65-74-women	-0.526**	0.627**
	≥75-men	-0.526**	0.861**
	≥75-women	-0.526**	0.823**
SHDI	Men	0.599**	0.752**
	Women	0.599**	1.033**
	15-64	0.599**	0.295*
	65-74	0.599**	0.490*
	≥75	0.599**	0.970**
	15-64-women	0.599**	0.554**
	65-74-men	0.599**	0.666**
	≥75-men	0.599**	0.869**
	≥75-women	0.599**	1.027**

* $p<0.05$** $p<0.01$

Table S13. Results of Granger causality test between ESs and HWMI.

Null hypothesis (H_0)	F-value	p-value
Biodiversity is not the Granger causality of HWMI	4.57	0.043*
Cultural service is not the Granger causality of HWMI	9.187	0.006**
Water supply service is not the Granger causality of HWMI	0.347	0.562
Carbon sequestration service is not the Granger causality of HWMI	9.371	0.006**
Cooling service is not the Granger causality of HWMI	6.199	0.020*

* $p<0.05$; ** $p<0.01$
